# Registered report: Social face evaluation: ethnicity-specific differences in the judgement of trustworthiness of faces and facial parts

**DOI:** 10.1038/s41598-022-22709-9

**Published:** 2022-10-31

**Authors:** Irina Schmid, Zachary Witkower, Friedrich M. Götz, Stefan Stieger

**Affiliations:** 1 Department of Psychology and Psychodynamics, Karl Landsteiner University of Health Sciences, Krems an der Donau, Austria; 2 Department of Psychology, University of Toronto, Toronto, Canada; 3 Department of Psychology, University of British Columbia, Vancouver, Canada; 4 Institute of Personality and Social Research, University of California, Berkeley, USA

**Keywords:** Social evolution, Human behaviour

## Abstract

**Abstract:**

Social face evaluation is a common and consequential element of everyday life based on the judgement of trustworthiness. However, the particular facial regions that guide such trustworthiness judgements are largely unknown. It is also unclear whether different facial regions are consistently utilized to guide judgments for different ethnic groups, and whether previous exposure to specific ethnicities in one’s social environment has an influence on trustworthiness judgements made from faces or facial regions. This registered report addressed these questions through a global online survey study that recruited Asian, Black, Latino, and White raters (*N* = 4580). Raters were shown full faces and specific parts of the face for an ethnically diverse, sex-balanced set of 32 targets and rated targets’ trustworthiness. Multilevel modelling showed that in forming trustworthiness judgements, raters relied most strongly on the eyes (with no substantial information loss vis-à-vis full faces). Corroborating ingroup–outgroup effects, raters rated faces and facial parts of targets with whom they shared their ethnicity, sex, or eye color as significantly more trustworthy. Exposure to ethnic groups in raters’ social environment predicted trustworthiness ratings of other ethnic groups in nuanced ways. That is, raters from the ambient ethnic majority provided slightly higher trustworthiness ratings for stimuli of their own ethnicity compared to minority ethnicities. In contrast, raters from an ambient ethnic minority (e.g., immigrants) provided substantially lower trustworthiness ratings for stimuli of the ethnic majority. Taken together, the current study provides a new window into the psychological processes underlying social face evaluation and its cultural generalizability.

**Protocol registration:**

The stage 1 protocol for this Registered Report was accepted in principle on 7 January 2022. The protocol, as accepted by the journal, can be found at: 10.6084/m9.figshare.18319244.

## Introduction

Social perception, which is how people form impressions of and make inferences about others, is a fundamental feature of human interactions that influences our social behavior in multiple ways: it guides our decisions about who is safe to approach or avoid, whom we should befriend, and whom we can follow, trust, and learn from. Hence, there has been great scientific interest in investigating how humans judge faces^1–3^.

One of the most popular models of social face evaluation (1747 citations on Google Scholar as of May 25th, 2022) was developed by Oosterhof and Todorov^4^ who used bottom-up methods to uncover two orthogonal (i.e., independent) factors that broadly capture trait judgments of expressively neutral faces: valence (referring to whether someone should be avoided or can be approached safely) and dominance (referring to physical strength and weakness). In this study, 327 raters judged neutral European faces along 15 different personality traits. A principal components analysis showed that the valence factor explained a large proportion (63.3%) of the variance in the ratings, and that judgments of trustworthiness can be used as an approximation of this underlying dimension. From an evolutionary vantage point, the valence (trustworthiness) component may communicate information that is critical for survival, such as whether or not someone harbors harmful intentions^4^. In other words, humans have a strong incentive to correctly assess the trustworthiness of individuals, including from their face. This then raises the equally fundamental and complex question: *How* do humans judge trustworthiness from looking at someone else’s face?

The current research pursues two broad research goals. In light of the scientific literature reviewed above, its first goal is to elucidate the mechanics of human trustworthiness judgements. To that end it raises and addresses the following research question (RQ1): *Is the judgement of trustworthiness of singular facial parts (eyes/mid-face/mouth) different from the judgement of trustworthiness of whole faces?* Beyond illuminating which (if any) specific areas of a face humans use to guide perceptions of trustworthiness, as a second research goal the current study also seeks to examine how these perceptual processes are qualified by the ethnicity of the faces being judged as well as the ethnicity of those judging them. More specifically, we investigate whether—and if so to which extent—humans vary in the facial cues that guide their judgments of trustworthiness based on the targets’ ethnicity and perceivers’ (i.e., raters’) ethnicity (RQ2).

Regarding RQ1, a large body of research, including work on the face-inversion effect^5^ and the composite face illusion^6^, provides empirical evidence that faces are perceived holistically^7^. Yet, a growing number of studies have demonstrated the importance of specific, individual facial features in forming perceptions of neutral faces^8–13^. For example, it was found that changing the appearance of the eyebrows can have a large effect on perceptions of threat—a construct inversely related to trustworthiness^14^. Furthermore, previous research showed that varying the size of the eyes directly impacts perceptions of trustworthiness, such that individuals with larger eyes are perceived as more trustworthy^14^. In light of these findings, it appears plausible that humans evaluate trustworthiness, at least in part, on the basis of particular facial regions. However, it is less clear which specific facial regions are used to guide perceptions of trustworthiness.

There is robust evidence indicating that when forming judgments of trustworthiness from a person’s face, people rely on their natural tendency to infer *emotion* from the face^15^. According to Basic Emotion Theory^16^, humans evolved several universal “basic” emotions (i.e., fear, happiness, anger, surprise, disgust, and sadness), which were naturally selected^17–21^. Each emotion is associated with a distinct, readily-interpretable, and universal facial expression, suggesting that the ability to accurately infer basic emotions from emotionally expressive faces is genetically hard-wired^19,22^ (but see^23–25^ for evidence against this proposition). In fact, humans are so sensitive to the communication of emotion via the face that they overgeneralize emotion perception to form judgments of *expressionless* (i.e., neutral and resting) faces—a process called emotion overgeneralization^15,26^. For example, faces that structurally resemble emotion expressions are perceived to be more characteristic of that emotion (e.g., resting faces with slightly upturned lip corners are perceived as happier, whereas resting faces with lower eyebrows are perceived as angrier^27^). These naturally-occurring emotion-laden perceptions are, in turn, critical for guiding perceptions of trustworthiness. Indeed, one study found that perceptions of happiness, fear, and (low) perceptions of anger formed from neutral faces are the strongest predictors of trustworthiness judgments formed from expressionless faces^20^.

Critically, humans are able to recognize all basic emotions from expressive faces when solely viewing someone’s eyes with the rest of the face occluded^28,29^. Given that emotion perception is foundational to guiding perceptions of trustworthiness from expressionless faces, perceivers might rely on this critical facial region to similarly guide their perceptions of trustworthiness. Consistent with this, participants direct their visual attention to the eyes of targets in order to guide their judgments of faces^30^. In fact, one diagnostic tool to assess theory of mind and mentalizing, the *Reading the Mind in the Eyes Test*^29^, works under the assumption that neurotypical individuals are able to form accurate impressions of others from their eyes and eyebrows^29^. Therefore, it is possible that humans will chiefly rely on the eye region of a face to form their perceptions of trustworthiness.

A second possibility is that adults rely on the mouth and chin to guide their evaluations of trustworthiness^31,32^. Corroborating this notion, there is scientific evidence that the mouth region might play an important role in scanning emotional face expressions, especially for happy faces^33,34^. The chin and jaw are sexually dimorphic features (i.e., they provide diagnostic information about the sex of the person) that guide ascriptions of masculinity and dominance—traits associated with trustworthiness^35–37^. Furthermore, the facial region around the mouth contains the most facial muscles, which makes it the most variable and differentiated^38^, possibly containing useful information to guide trustworthiness perceptions.

A final possibility is that participants require the whole face to guide their perceptions. For example, individuals might rely on holistic face structures—such as the facial width-to-height ratio—to guide their perceptions of trustworthiness^39^. Occluding any one part of the face disables an individual’s ability to use the facial width-to-height ratio to guide their judgments, given that the facial width and facial height are not visible in their entirety. Alternatively, raters might have an internal representation of what a trustworthy face looks like, and occluding parts of the face could disrupt their ability to compare that face to their preconceived template; occluding parts of the face might therefore be necessary for an observer to form differentiated perceptions of trustworthiness from faces^40–42^.

Turning to RQ2, surveying the extant literature renders the possibility of ethnicity-specific perceptual effects likely. For example, a multi-site study from the *Psychological Science Accelerator* (PSA) initiative^43^ was generally able to replicate the original findings of Oosterhof and Todorov^4^ across 11 world regions and 41 countries in ethnically diverse stimuli—including the central role of valence/trustworthiness in social face evaluations. However, model fit for the valence-dominance model differed significantly across world regions, with diminished fit in Asian countries, alluding to the possibility of systematic ethnicity-based perceptual differences^44^. Other studies further lend support to this assumption. White raters spend more time attending to the eyes of other White faces than Black faces^45,46^, and Asian faces^47,48^. This attention bias has important implications for emotion recognition: attention to the eyes predicts accuracy in happiness ratings made from prototypical happiness expressions. As a result, reduced attention to the eyes of black targets predicts White raters’ deficits in recognising happiness expressions on Black faces^46^. Moreover, recent research showed that face scanning during dyadic social interactions is modulated by culture as, for example, Japanese raters show increased scanning activity in the central face and eye region, whereas British/Irish raters tend to focus on the mouth region^49^. Analogously, in the case of trustworthiness judgements, raters’ reliance on any particular facial feature might not be uniform across the ethnicities of faces.

One potential explanation for such divergent patterns might be perceptual ingroup biases. In general, people perceive individuals from one’s own group (e.g., ethnic groups, kinship) as more trustworthy than people from other groups^50^. This might also be reflected in different perception strategies. That is, the eyes might be less critical for perceptions of trustworthiness formed of outgroup members versus ingroup members. Instead, when forming perceptions of trustworthiness of outgroup members, parts of the face *besides* the eyes—including the nose or the mouth, might be more important. Although past research has demonstrated that raters judge targets from their own ingroup differently than targets from an outgroup^40^, research has yet to look at how ingroup status guides perceptions of trustworthiness for specific facial parts. To formally address this, in the current research we pose and empirically investigate RQ2A. *Do humans judge the trustworthiness of faces/facial parts of the stimuli from their own ethnicity differently compared to stimuli from other ethnicities?*

Relatedly, in addition to a target’s ethnicity, the ethnicities a person perceives most frequently and in the highest quantity in their social environment could also guide their trustworthiness perceptions, and the perceptual mechanisms that observers use to guide these perceptions. Recent experiences with other faces can substantially impact how we perceive faces (i.e., “after-effects”, “also: “mere-exposure effects”^51^); for example, by changing individuals’ mental representations of what constitutes an average face—and in turn the ways in which each specific face may deviate from this norm (including facial features like skin color^52^). As the typical or dominant skin color, hair color, eye color etc. vary among ethnicities, exploring ethnicity-specific adaption in face perception—including the influence of the dominant ethnicities of one’s social environment—is of great interest to get a more complete understanding of the processes at hand, as captured in RQ2B*. Do humans judge the trustworthiness of faces/facial parts of stimuli from the dominant ethnicity of their social environment differently compared to stimuli from other ethnicities?* Taking into consideration that the dominant ethnicity of one’s social environment is not always identical with one’s own ethnicity, RQ2B contributes to examining the ingroup preference assumption in a more nuanced way by attending to whether raters belong to an ethnic majority or minority in their social environment.

In summary, the proposed study advances our understanding of how people evaluate trustworthiness from faces by empirically investigating: (1) whether—and if so to which extent—humans rely on specific facial features (as opposed to holistic face perceptions) when forming trustworthiness judgements and (2) how the reliance on these differential facial cues differs across target ethnicities, and whether any such perceptual differences are a function of prior exposure to faces of the respective ethnicities (see Table 1). In addition, as a purely exploratory set of analyses, we will test for potential moderating effects of target sex (explorative analysis 1: *EA1*), rater sex, eye color, and hair color (*EA2*) and the difficulty to do the judgements (*EA3*) on the perception strategies that raters employ to judge trustworthiness. EA1 is included because the sex of the target might have a significant impact on the shape and anatomy of the face (e.g., chin) and therefore on the crucial facial structures for face evaluation. Furthermore, EA2 investigates whether there are any self-identification bias effects (e.g., are targets with the same sex/eye color/hair color as the rater rated more trustworthy?) and EA3 takes the difficulty of trustworthiness judgements into account.

**Table 1:** Study design overview.

Question	Hypothesis (if applicable)	Sampling Plan (e.g., power analysis)	Analysis Plan	Interpretation given to different outcomes
*RQ1. Is the judgement of trustworthiness of singular facial parts (eyes/ mid-face /mouth) different from the judgement of trustworthiness of whole faces?*	H0: Trustworthiness judgements of singular facial parts are not different from trustworthiness judgements of the whole face H1: Trustworthiness judgements of singular facial parts are different from trustworthiness judgements of the whole face	α = 5%, minimum power = 99%, two-sided; ICC = 0.30; 15% non-response and dropout rate), needed sample size is *N* = 2276 raters^53^	Random-effects multilevel model: L1 (within-person) = ratings of different faces/facial parts; L2 (between-person) = raters	If trustworthiness judgements of one or more facial parts do not significantly differ (*p* ≥ 0.05) from the whole face (i.e., reference category), then this/these facial part/s is/are primarily responsible for the trustworthiness judgments of faces
*RQ2A. Do humans judge the trustworthiness of faces/facial parts of the stimuli from their own ethnicity differently compared to stimuli from other ethnicities?*	H0: Humans do not judge the trustworthiness of faces/facial parts of the stimuli from their own ethnicity differently compared to stimuli from other ethnicities H1: Humans judge the trustworthiness of faces/facial parts of the stimuli from their own ethnicity differently compared to stimuli from other ethnicities	α = 5%, minimum power = 99%, two-sided; ICC = 0.30; 15% non-response and dropout rate), needed sample size is *N* = 2276 raters^53^	Random-effects multilevel model: L1 (within-person) = ratings of different faces/facial parts; L2 (between-person) = raters	If one of the rater ethnicities is significant (*p* < 0.05), this means that raters judge the trustworthiness of whole faces/facial parts differently depending on whether target’s ethnicity matches/mismatches their own ethnicity If none of the rater ethnicities are significant (*p* ≥ 0.05), this means that raters judge the trustworthiness of whole faces/ facial parts independent of whether target’s ethnicity matches/mismatches their own ethnicity
*RQ2B. Do humans judge the trustworthiness of faces/facial parts of stimuli from the dominant ethnicity of their social environment differently compared to stimuli from other ethnicities?*	H0: Humans do not judge the trustworthiness of faces/facial parts of stimuli from the dominant ethnicity of their social environment differently compared to stimuli from other ethnicities H1: Humans judge the trustworthiness of faces/facial parts of stimuli from the dominant ethnicity of their social environment differently compared to stimuli from other ethnicities	α = 5%, minimum power = 99%, two-sided; ICC = 0.30; 15% non-response and dropout rate), needed sample size is *N* = 2276 raters^53^	Random-effects multilevel model: L1 (within-person) = ratings of different faces/facial parts; L2 (between-person) = raters	If the rater’s dominant ambient ethnicity is significant (*p* < 0.05), this means there is a difference between raters’s dominant ambient ethnicity and other ethnicities regarding the judgements of trustworthiness of whole faces and facial parts If the rater’s dominant ambient ethnicity is not significant (*p* ≥ 0.05), this means there is no difference between the rater’s dominant ambient ethnicity and other ethnicities regarding the judgements of trustworthiness of whole faces and facial parts
*EA1: target sex*	Not applicable	α = 5%, minimum power = 99%, two-sided; ICC = 0.30; 15% non-response and dropout rate), needed sample size is *N* = 2276 raters^53^	Random-effects multilevel model: L1 (within-person) = ratings of different faces/facial parts; L2 (between-person) = raters	
*EA2: rater sex, eye color, and hair color*	Not applicable	α = 5%, minimum power = 99%, two-sided; ICC = 0.30; 15% non-response and dropout rate), needed sample size is *N* = 2,276 raters^53^	Random-effects multilevel model: L1 (within-person) = ratings of different faces/facial parts; L2 (between-person) = raters	
*EA3: difficulty of rating the stimuli of the full faces, eyes parts, mid-face parts, and mouth parts*	Not applicable	α = 5%, minimum power = 99%, two-sided; ICC = 0.30; 15% non-response and dropout rate), needed sample size is *N* = 2,276 raters^53^	Random-effects multilevel model: L1 (within-person) = ratings of different faces/facial parts; L2 (between-person) = raters	

To that end, the current project draws from a large, ethnically diverse sample of raters, who completed an online questionnaire available in five languages (English, German, Spanish, Mandarin, Japanese).

## Methods

### Design

#### Recruitment

Raters were recruited via the online participant database *Prolific* (https://www.prolific.co/; for previous research demonstrating the utility of and data quality afforded by *Prolific* see^54,55^). Raters received a numeration upon completion of the study, which took approximately 15 min according to pre-tests.

*Prolific* offers several filtering options for rater recruitment, including filters on *ethnicity*. In Table 2, we exhibit the ethnic categories which were used for participant filtering in *Prolific*.

**Table 2:** Prolific ethnicity categories.

Ethnic categories of study	Chosen ethnic categories on Prolific
Asian	East Asian, South Asian, South-East Asian
Black	African, Black/African American, Black/British
Latino^a^	Latino/Hispanic, White Mexican
White	White/Caucasian, White/Sephardic Jew

^a^As the stimuli of the study derive from the Chicago Face Database (https://chicagofaces.org/default/), we use “Latino” in order to provide consistent terms.

Moreover, we applied a filter on nationality to ensure that we would only recruit raters from nations where the official language is one of the five languages that our questionnaire was available in. Therefore, we filtered for the following nations (filter *nationality*; see Table 3).

**Table 3:** Nationality filter on *Prolific*.

Language version	Nation(s)
English	Australia, Bahamas, Canada, India, Ireland, Jamaica, South Africa, United Kingdom, United States
German	Austria, Germany, Liechtenstein, Switzerland
Japanese	Japan
Mandarin	China
Spanish	Argentina, Bolivarian Republic, Chile, Costa Rica, Cuba, Dominican Republic, Ecuador, El Salvador, Honduras, Mexico, Panama, Puerto Rico, Spain, Uruguay, Venezuela

To ensure appropriate filtering, we additionally asked for raters’ ethnicity in our online questionnaire. As such, we checked whether the self-reported ethnicity in *Prolific* matched the reported ethnicity.

#### Data collection

**Figure 1:**
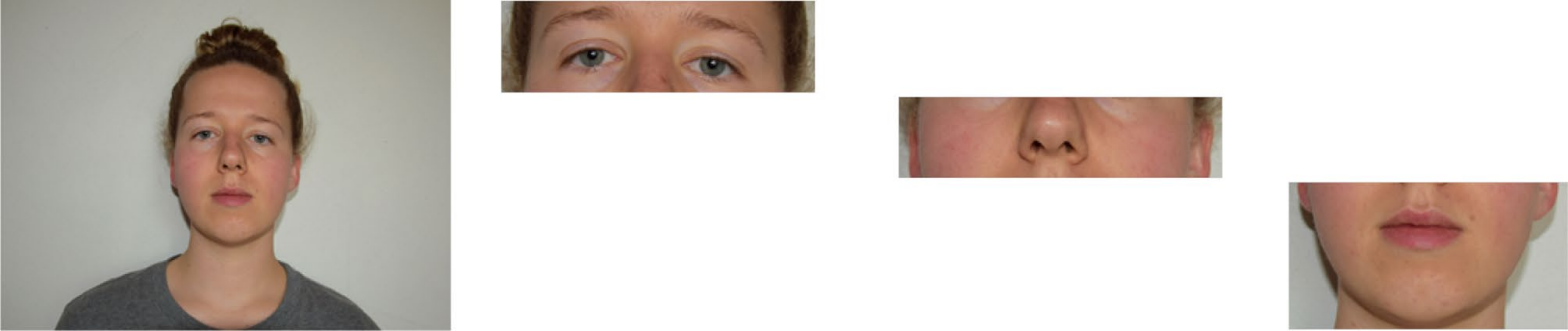
Example of stimuli: whole face (far left), eyes part (middle left), middle-face part (middle right), and mouth part (far right). Consent for publication has been given.

An online questionnaire (5 language versions: English, German, Japanese, Mandarin, and Spanish) was used for data collection. After agreeing to an Informed Consent and providing socio-demographic and biometric information (sex, age, years of education, primary country of residence, ethnicity, predominant ethnicity of social environment, hair color, eye color, nationality), raters were instructed to rate 32 different faces (see “Materials” for more information on these faces). The stimuli were displayed in four different versions: full face, eyes part, middle-face part, mouth part. Stimuli in the eyes part condition were cropped between the crown and nasal bone. Stimuli in the mid-face condition (notably encompassing the nose and the ears) were cropped between the nasal bone and at the height of the intermaxillary suture. Stimuli in the mouth part condition were cropped between the intermaxillary suture and mandible (see Fig. 1).

**Figure 2:**
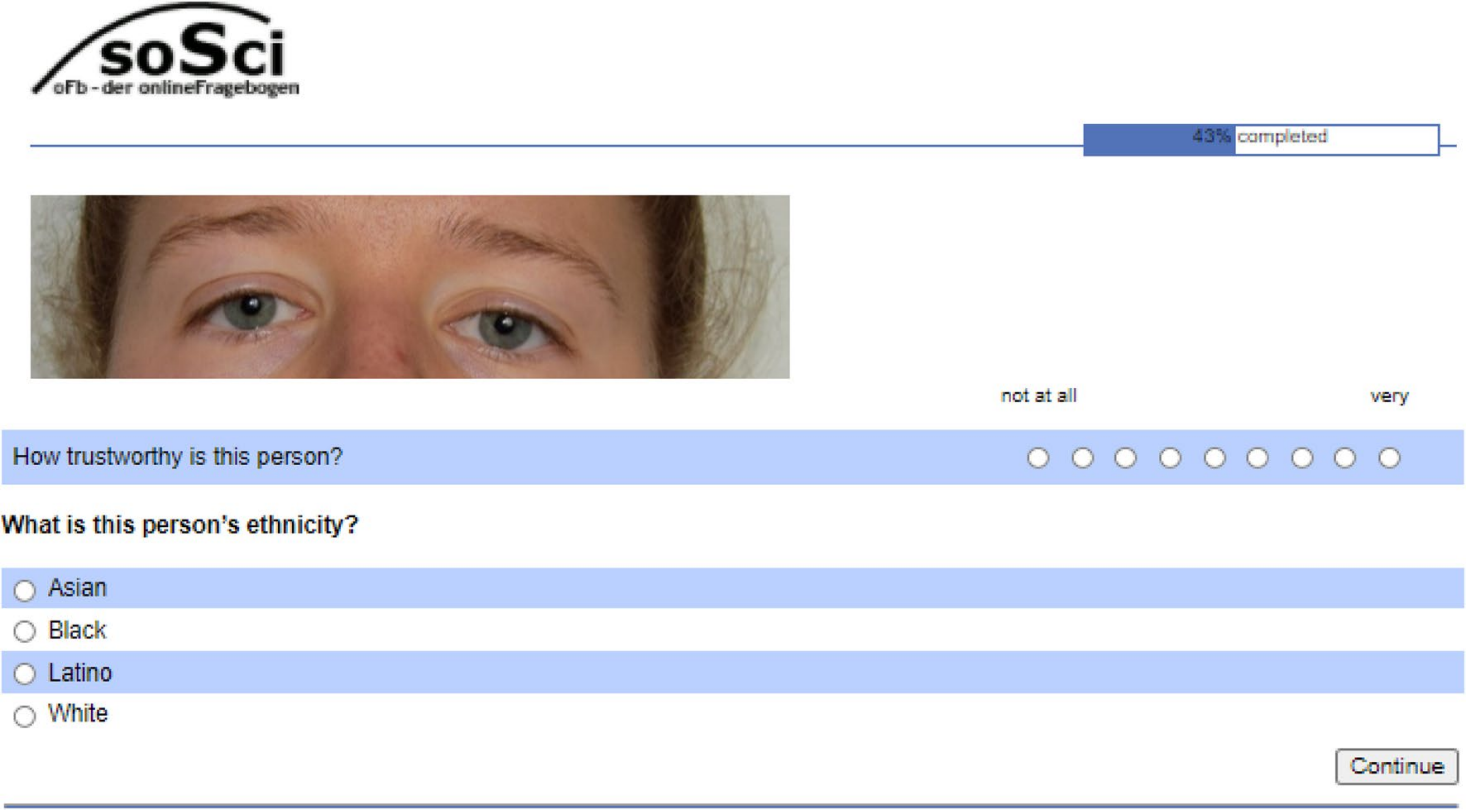
English example task.

Raters judged the trustworthiness of each randomly ordered image, as spontaneously as possible, using a scale from 1 (*not at all*) to 9 (*very*). The stimuli were presented as one block; that is, participants rated 128 (64 female, 64 male; 32 Asian, 32 Black, 32 Latino, 32 White) faces/face parts in terms of their trustworthiness (“How trustworthy is this person?”; cf.^48^), in a randomized order. To control for the identification of the correct ethnicity of stimuli, raters were asked about the perceived ethnicity of the target (possible answers: Asian, Black, Latino, White). In addition, raters were asked once at the end of the questionnaire how difficult they generally found it to rate the stimuli (see EA3) of the full faces and each facial part (scale: 1 = *not at all*, 9 = *very*). For an illustration, see Fig. 2.

#### Equipment

A computer with a stable Internet connection and a user account on the crowd-sourcing platform *Prolific* was required to access the online study.

#### Materials

The materials used included sex-balanced sets of images (i.e., target stimuli) consisting of 8 Asian, 8 Black, 8 Latino and 8 White faces (4 male, and 4 female faces for each of the 4 ethnicity categories). Each face was presented in four different stimulus types, as described in Fig. 1 (i.e., eyes only, middle-face only, mouth only, and whole face). All images were taken from the Chicago Face Database (CFD; https://chicagofaces.org/default/). As outlined by Ma et al.^56^, the photographs were taken under standardized conditions, where targets wore a grey T-shirt and looked directly into the camera. The models had a neutral facial expression and were photographed against a white background (see Fig. 1). All targets used in the current study were between the ages of 25–35 years and were accurately recognized as their self-reported ethnicity > 60% of the time in the CFD norming data. We selected targets who were recognized as their self-reported ethnicity > 60% of the time, as we expected perceivers (i.e., raters) to be less likely to exhibit outgroup biases if they cannot accurately identify a member as an outgroup. For the stimuli that only display one facial part, the original images were cropped. Furthermore, Ma et al.^56^ had participants rate how trustworthy each target was “with respect to other people of the same race and gender”. On that scale, the 32 targets used in the current study were rated as moderately trustworthy (*M* = 3.68, *SD* = 0.32, min = 3.04, max = 4.27).

#### Translations

In line with past translational procedures from large cross-cultural, multi-lab projects^44^, the following translational steps were performed.

Step 1 (Translation). The original version of the online questionnaire (including Informed Consent) was translated from English to the target language by either one of the authors or academics who are fluent in the target language (German, Spanish, Japanese, and Mandarin), resulting in Version A.

Step 2 (Back-translation). Version A was then translated back from the target language to English by either one of the authors or academics who are fluent in the target language, resulting in Version B.

Step 3 (Discussion). Both versions were discussed by the authors and/or academics who are fluent in the target language in order to check for discrepancies and find solutions to them, resulting in Version C.

Step 4 (External readings). Version C was tested by individuals who are fluent in the target language but were not involved in the translation procedure during the previous three steps. Any possible misunderstandings and necessary adjustments were noted, discussed, and implemented, resulting in the final Version D.

### Sampling

#### Statistical power analysis

Based on the results of Taubert et al.^5^ we expected a small effect size (*f* = 0.10) for all research questions. Power analyses for multi-level designs are usually calculated based on a pre-test or during the data collection because many different parameters have to be estimated^54^. Fortunately, there are procedures to roughly estimate the needed sample size a priori such as the method introduced by Twisk^53^. On the basis of this method (α = 5%, minimum power = 99%, two-sided; assumed ICC = 0.30; 128 observations), the estimates required sample size to detect a small effect (*f* = 0.10) was *N* = 569 raters. Because this represents only a rough estimate and we also focused on interaction effects which usually require larger samples, we aimed for *N* = 1000 raters per ethnicity.

#### Eligibility criteria

Raters’ minimum age was set at 18 years and all raters had to provide an Informed Consent. Additionally, raters were required to have normal or corrected-to-normal vision (e.g., wearing glasses/contact lenses). Raters with invariant response patterns (i.e., those who rated more than 75% of faces identically) as well as raters who did not finish the study or skipped too many stimuli, (i.e., those who had more than 25% missings), were excluded from further analyses. In case of missing trustworthiness ratings of less than 25%, the mean rating over all raters for the respective stimulus was inserted (i.e., item mean substitution; see^58^) but this was only necessary for a few cases (*n*_missing_ = 2 [0.1%]–19 [0.6%]). There were no further exclusion criteria based on other person-related variables (e.g., sex, sexual orientation, religious beliefs).

#### Raters

To examine the research questions outlined above, data from raters across four different ethnicities (Asian, Black, Latino, White) were collected (*note*: without nationality quotas). The total sample included *N*_total_ = 4580 raters, of which 2 raters were excluded because they stated to be younger than 18 years. Moreover, raters who did not finish the study (*n* = 343), raters with more than 25% missings (*n* = 16) or with invariant response patterns (*n* = 848), were not considered in further analyses.

Our final sample consisted of *N*_final_ = 3371 raters (63.1% female, 36.0% male, 0.7% other, 0.2% no answer; *M*_age_ = 30.5, *SD*_age_ = 11.1, range = 18–84 years). Concerning formal educational attainment, the average number of completed education years was *M*_education_ = 14.6 years (*SD*_education_ = 4.7, median = 16, mode = 16, range = 1–25 years).

As we collected data from raters across four different ethnicities (*note*: without nationality quotas) the final sample included 22.0% Asian raters, 26.9% Black raters, 21.7% Latino raters, 26.4% White raters, and 2.8% raters of mixed ethnicity. A third of raters stated to have a predominantly White social environment, the other two thirds considered their social environment predominantly Black (24.7%), Latino (17.4%), Asian (11.9%) or mixed/not clearly classifiable (12.9%). Geographically, the study sample was widely spread across 32 different countries, whereby raters predominantly came from South Africa, the UK, and the USA. For further details on the demographic composition of the sample see Figure S1 in the Online Supplement (accessible via https://osf.io/tcyqs/).

With regard to raters’ eye color, the vast majority was brown-eyed. Concerning natural hair color, the most common hair colors were black and brown, while other hair colors were less frequent. More detailed sample characteristics can be found in Table S1 of the Online Supplement.

### Statistical analyses

We used SPSS version 27 for the statistical analyses of Step 1–3. All calculations of the main analyses were conducted using *R*^59^ in combination with the *lme4*^60^, *lmerTest*^61^, and *sjstats* packages^62^. For standardized coefficients, we used the *effectsize* package^63^ which takes the different levels of standardization into account. That is, level 1 parameters are standardized within groups, while level 2 parameters are standardized between groups^64^.

For data analysis, we carried out the following four steps.

Step 1: Checking inclusion criteria and data quality.

Raters younger than 18 years of age and raters who did not finish the study were excluded from further analyses. By inspecting the frequencies of selected rating categories for each individual participant identified 848 raters (These raters were characterized by consistently selecting one of the extremes (i.e., 1, 9) or the middle category (i.e., 5) of the rating scale combined with exceedingly fast completion times, indicating response tendency biases due to low involvement/participant motivation (e.g.,^67^).) (18.5%) with invariant response patterns (i.e., identical ratings for more than 75% of the stimuli) which were subsequently excluded from analysis, in line with our predetermined exclusion criteria. Moreover, 16 raters who had more than 25% missings and trials with misidentified target ethnicity were not considered for further statistical analyses. If trustworthiness ratings were missing for less than 25%, the mean rating over all raters for the respective stimulus was inserted.

Step 2: Descriptive statistics.

Descriptive analyses were conducted for raters’ age, sex, educational level, ethnicity, ethnicity of social environment, hair color, eye color, and the difficulty of ratings.

Step 3: Main analyses for RQ1 and RQ2.

Random-intercept, random-slope multi-level regression models were fitted to examine the effects of facial parts (whole, mouth, mid-face, eyes; RQ1), target sex (EA1), match between participants’ sex and target sex, match in eye color and hair color (EA2), as well as general difficulty of ratings (EA3) on trustworthiness ratings of the depicted targets in the stimuli pictures. Furthermore, for RQ2, we analyzed ethnicity matches between raters and targets as well as differences in trustworthiness depending on (mis-)matching ethnicity of targets and the dominant ethnicity in raters’ social environment. Multi-level models account for the nested design of our study with different stimulus pictures (level 1) nested within raters (level 2). All level 2 predictors were grand-mean centered except for rater’s sex and ethnicity^65,66^.

We first ran a baseline model without any predictors to calculate intraclass correlation coefficient (ICC) values. ICC of the null-model was 29%, neatly aligning with the assumptions of our power analysis (assumed ICC = 30%). Next, we ran random-intercept random-slope models and random-intercept fixed-slope models as described below. Because the random-intercept random-slope model fitted the data better for RQ1 (χ^2^ = 9317.9, *df* = 9, *p* < 0.001), all subsequent models were run with this specification. Of note, the model fitted for RQ1 (as well as EA1, EA2, and EA3) did not converge. Therefore, we excluded random effects for the match variables (Match—sex, Match—eye color, Match—hair color) to reach convergence.

The model employed to examine RQ1 can be formalized as follows (i.e., Step 1 in Table 4):

$$\begin{aligned} & {\text{Level 1 }}\left( {\text{within person}} \right){:}{\text{ Trustworthiness}}_{{{\text{ti}}}} = \, \pi_{{0{\text{i}}}} + \, \pi_{{{\text{1i}}}} {\text{ Mouth part}}_{{{\text{ti}}}} + \, \pi_{{{\text{2i}}}} {\text{ Nose part}}_{{{\text{ti}}}} + \, \pi_{{{\text{3i}}}} {\text{ Eyes part}}_{{{\text{ti}}}} + e_{{{\text{ti}}}} \\ & {\text{Level 2 }}\left( {\text{between persons}} \right){:} \, \pi_{{{\text{1i}}}} = \, \beta_{{{1}0}} + r_{{{\text{1i}};}} \pi_{{{\text{2i}}}} = \, \beta_{{{2}0}} + r_{{{\text{2i}};}} \pi_{{{\text{3i}}}} = \, \beta_{{{3}0}} + r_{{{\text{3i}}}} \\ \end{aligned}$$

**Table 4:** Trustworthiness assessments of whole face (reference) and different facial parts (RQ1).

	Fixed						Random	
	Coeff	*B*	*CI*	*Stand. B*	*SE*	*t*	Coeff	*SD*
**Step 1: RQ1**								
Intercept (Reference)	β_00_	5.42	5.39 to 5.46		0.02	292.6***	*r*_0*i*_	1.04
Within-person (reference whole face)								
Mouth part	β_10_	− 0.38	− 0.41 to − 0.36	− 0.10	0.01	− 30.9***	*r*_1*i*_	0.60
Mid-face part	β_20_	− 0.41	− 0.43 to − 0.38	− 0.11	0.01	− 29.2***	*r*_2*i*_	0.71
Eyes part	β_30_	> − 0.01	− 0.02 to 0.02	> − 0.01	0.01	− 0.10	*r*_3*i*_	0.46
*N*_observations_ = 431,488; *N*_raters_ = 3,371; *R*^2^_conditional_ = 33%, AIC = 1,634,392, BIC = 1,634,557, Ω^2^ = 34%								
**Step 2: RQ1 + EA1 + EA2 + EA3**								
Intercept (Reference)	β_00_	5.26	5.21 to 5.30		0.02	252.4***	*r*_0*i*_	1.08
Within-person (reference whole face)								
Mouth part	β_10_	− 0.38	− 0.41 to − 0.36	− 0.10	0.01	− 30.8***	*r*_1*i*_	0.62
Mid-face part	β_20_	− 0.41	− 0.44 to − 0.38	− 0.11	0.01	− 29.1***	*r*_2*i*_	0.72
Eyes part	β_30_	> − 0.01	− 0.02 to 0.02	> − 0.01	0.01	− 0.1	*r*_3*i*_	0.47
Target-sex (male)	β_40_	− 0.43	− 0.45 to − 0.42	− 0.13	0.01	− 44.1***	*r*_4*i*_	0.48
Match—sex	β_50_	0.09	0.07 to 0.11	0.03	0.01	9.2***		
Match—eye color	β_60_	0.59	0.58 to 0.61	0.18	0.01	95.7***		
Match—hair color	β_70_	− 0.09	− 0.10 to − 0.08	− 0.03	0.01	− 16.6***		
**Between-subject**								
Difficulty face	β_01_	< 0.01	− 0.02 – 0.02	> − 0.01	0.01	0.1		
Difficulty mouth	β_02_	− 0.01	− 0.03 to 0.01	− 0.03	0.01	− 1.3		
Difficulty mid-face	β_03_	0.04	0.02 to 0.06	0.08	0.01	3.5***		
Difficulty eyes	β_04_	> − 0.01	− 0.02 to 0.02	> − 0.01	0.01	− 0.3		
*N*_observations_ = 427,136; *N*_raters_ = 3,337; *R*^2^_conditional_ = 39%, AIC = 1,591,754, BIC = 1,592,061, Ω^2^ = 39%								

Whole face trust ratings served as reference category; *p < 0.05, **p < 0.01, ***p < 0.001.

The model employed to examine RQ1 including EA1-EA3, can be formalized as follows (i.e., Step 2 in Table 4):

$$\begin{aligned} & {\text{Level 1 }}\left( {\text{within person}} \right){:} {\text{ Trustworthiness}}_{{{\text{ti}}}} = \, \pi_{{0{\text{i}}}} + \, \pi_{{{\text{1i}}}} \; {\text{ Mouth part}}_{{{\text{ti}}}} + \, \pi_{{{\text{2i}}}} {\text{ Nose part}}_{{{\text{ti}}}} + \, \pi_{{{\text{3i}}}} \; {\text{ Eyes part}}_{{{\text{ti}}}} + \, \pi_{{{\text{4i}}}} {\text{ Target sex}}_{{{\text{ti}}}} + \, \pi_{{{\text{5i}}}} {\text{ Match sex}}_{{{\text{ti}}}} + \, \pi_{{{\text{6i}}}} {\text{ Match eyecolor}}_{{{\text{ti}}}} + \, \pi_{{{\text{7i}}}} {\text{ Match haircolor}}_{{{\text{ti}}}} + e_{{{\text{ti}}}} \\ & {\text{Level 2 }}\left( {\text{between persons}} \right){:} \, \pi_{{0{\text{i}}}} = \, \beta_{00} + \, \beta_{{0{1}}} {\text{ Difficulty face}}.{\text{cgm}}_{{\text{i}}} + \, \beta_{{0{2}}} {\text{ Difficulty mouth}}.{\text{cgm}}_{{\text{i}}} + \, \beta_{{0{3}}} {\text{ Difficulty nose}}.{\text{cgm}}_{{\text{i}}} + \, \beta_{{0{4}}} {\text{ Difficulty eyes}}.{\text{cgm}}_{{\text{i}}} + r_{{0{\text{i}}}} \\ & {\text{Level 2 }}\left( {\text{between persons}} \right){:} \pi_{{{\text{1i}}}} = \beta_{{{1}0}} + r_{{{\text{1i}};}} \beta_{{{2}0}} + r_{{{\text{2i}};}} \beta_{{{3}0}} + r_{{{\text{3i}};}} \beta_{{{4}0}} + r_{{{\text{4i}}}} \\ \end{aligned}$$

The model employed to examine RQ2A and RQ2B (with each face / facial part being considered separately) can be formalized as follows (see Tables 5, 6):

$$\begin{gathered} {\text{Level 1 }}\left( {\text{within person}} \right){:}{\text{ Trustworthiness}}_{{{\text{ti}}}} = \, \pi_{{0{\text{i}}}} + \, \pi_{{{\text{1i}}}} {\text{ Match ethnicity}}_{{{\text{ti}}}} + e_{{{\text{ti}}}} \hfill \\ {\text{Level 2 }}\left( {\text{between persons}} \right){:} \, \pi_{{0{\text{i}}}} = \, \beta_{00} + \, \beta_{{0{1}}} {\text{ Dominant ethnicity}}_{{\text{i}}} + r_{{0{\text{i}}}} \hfill \\ {\text{Level 2 }}\left( {{\text{between persons}},{\text{ interaction}}} \right){:}\pi_{{{\text{1i}}}} = \beta_{{{1}0}} + \beta_{{{11}}} {\text{ Match ethnicity}}_{{\text{i}}} *{\text{ Dominant ethinicity}}_{{\text{i}}} + r_{{{\text{1i}}}} \hfill \\ \end{gathered}$$

**Table 5:** Results of the multi-level analyses for RQ2A: mis-/matching rater and target ethnicity per stimulus type.

	Fixed						Random	
	Coeff	*B*	*CI*	*stand. B*	*SE*	*t*	Coeff	*SD*
**Whole face**								
Intercept	β_00_	5.39	5.35–5.43		0.02	275.1***	*r*_0*i*_	1.08
Match ethnicity	β_10_	0.15	0.11–0.19	0.04	0.02	7.8***	*r*_1*i*_	0.84
*R*^2^_conditional_ = 28%, AIC = 437,792, BIC = 438,850, Ω^2^ = 31%								
**Mouth part**								
Intercept	β_00_	5.02	4.98–5.06		0.02	250.9***	*r*_0*i*_	1.12
Match ethnicity	β_10_	0.10	0.06–0.13	0.03	0.02	5.7***	*r*_1*i*_	0.77
*R*^2^_conditional_ = 37%, AIC = 403,475, BIC = 403,532, Ω^2^ = 40%								
**Mid-face part**								
Intercept	β_00_	5.00	4.96–5.04		0.02	250.2***	*r*_0*i*_	1.13
Match ethnicity	β_10_	0.09	0.06–0.12	0.03	0.01	6.4***	*r*_1*i*_	0.60
*R*^2^_conditional_ = 45%, AIC = 369,850, BIC = 369,907, Ω^2^ = 47%								
**Eyes part**								
Intercept	β_00_	5.38	5.34–5.42		0.02	268.6***	*r*_0*i*_	1.12
Match ethnicity	β_10_	0.17	0.13–0.21	0.05	0.02	9.0***	*r*_1*i*_	0.90
*R*^2^_conditional_ = 35%, AIC = 414,294, BIC = 414,352, Ω^2^ = 38%								

Rater-target ethnicity match: 0 = mismatch; 1 = match. *N*_observations_ = 107,712; *N*_raters_ = 3,366; whole face trust ratings served as reference category. *p < 0.05, **p < 0.01, ***p < 0.001.

**Table 6:** Results of the multi-level analyses RQ2B: mis-/matching rater and social environment ethnicity per stimulus type including interactions.

	Fixed						Random	
	Coeff	*B*	*CI*	*Stand. B*	*SE*	*t*	Coeff	*SD*
**Whole face**								
Intercept (reference)	β_00_	5.42	5.39 – 5.46		0.02	276.9***	*r*_0*i*_	1.08
Dominant ethnicity match target (within)	β_10_	< 0.01	− 0.04 – 0.04	> − 0.01	0.02	0.04	*r*_1*i*_	0.96
**Whole face—including rater’s ethnicity and interaction effects**								
Intercept (reference)	β_00_	5.47	5.39 – 5.55		0.04	134.5***	*r*_0*i*_	1.08
Dominant ethnicity match target (within)	β_10_	− 0.78	− 0.89 to − 0.67	− 0.18	0.06	− 13.8***	*r*_1*i*_	0.91
Dominant ethnicity match rater (between)	β_01_	− 0.05	− 0.14 – 0.04	− 0.02	0.05	− 1.2		
Interaction	β_11_	0.90	0.78 – 1.02	0.20	0.06	14.8***		
*R*^2^_conditional_ = 28%, AIC = 437,766, BIC = 437,842, Ω^2^ = 31%								
**Mouth part**								
Intercept (reference)	β_00_	5.04	5.00 – 5.08		0.02	252.6***	*r*_0*i*_	1.12
Match ethnicity (within)	β_10_	− 0.01	− 0.05 – 0.03	> − 0.01	0.02	− 0.4	*r*_1*i*_	0.85
**Mouth part—including rater’s ethnicity and interaction effects**								
Intercept (reference)	β_00_	5.05	4.97 – 5.13		0.04	121.7***	*r*_0*i*_	1.12
Dominant ethnicity match target (within)	β_10_	− 0.57	− 0.67 to − 0.47	− 0.15	0.05	− 11.3***	*r*_1*i*_	0.82
Dominant ethnicity match rater (between)	β_01_	0.01	− 0.10 – 0.08	> − 0.01	0.05	− 0.2		
Interaction	β_11_	0.65	0.54 – 0.75	0.17	0.05	11.9***		
*R*^2^_conditional_ = 37%, AIC = 402,766, BIC = 402,842, Ω^2^ = 40%								
**Mid-face part**								
Intercept (reference)	β_00_	5.01	4.97 – 5.05		0.02	251.2***	*r*_0*i*_	1.13
Dominant ethnicity match target (within)	β_10_	0.02	− 0.01 – 0.05	> − 0.01	0.02	1.4	*r*_1*i*_	0.66
**Mid-face part**—**including rater’s ethnicity and interaction effects**								
Intercept (reference)	β_00_	5.06	4.98 – 5.14		0.04	121.9***	*r*_0*i*_	1.13
Dominant ethnicity match target (within)	β_10_	− 0.34	− 0.42 to − 0.26	− 0.11	0.04	− 8.3***	*r*_1*i*_	0.65
Dominant ethnicity match rater (between)	β_01_	− 0.06	− 0.15 – 0.04	− 0.02	0.05	− 1.2		
Interaction	β_11_	0.42	0.33 – 0.50	0.13	0.04	9.4***		
*R*^2^_conditional_ = 45%, AIC = 369,205, BIC = 369,281, Ω^2^ = 47%								
**Eyes part**								
Intercept (Reference)	β_00_	5.41	5.37 – 5.45		0.02	270.3***	*r*_0*i*_	1.12
Dominant ethnicity match target (within)	β_10_	0.05	0.00 – 0.09	0.01	0.02	2.1*	*r*_1*i*_	1.00
**Eyes part—including rater’s ethnicity and interaction effects**								
Intercept (Reference)	β_00_	5.50	5.41 – 5.58		0.04	132.2***	*r*_0*i*_	1.12
Dominant ethnicity match target (within)	β_10_	− 0.69	− 0.80 to − 0.58	− 0.18	0.06	− 12.3***	*r*_1*i*_	0.96
Dominant ethnicity match rater (between)	β_01_	− 0.11	− 0.20 to − 0.01	− 0.04	0.05	− 2.3*		
Interaction	β_11_	0.85	0.73 – 0.97	0.21	0.06	14.1***		
*R*^2^_conditional_ = 35%, AIC = 413,272, BIC = 413,348, Ω^2^ = 38%								

*N*_observations_ = 107,616; *N*_raters_ = 3,363; Reference category was the trust ratings of the whole face. *p < 0.05, **p < 0.01, ***p < 0.001.

The model employed to investigate potential cross-level interactions can be formalized as follows:

$$\begin{aligned} & & {\text{Level 1 }}\left( {\text{within person}} \right){:}{\text{ Trustworthiness}}_{{{\text{ti}}}} = \, \pi_{{0{\text{i}}}} + \, \pi_{{{\text{1i}}}} {\text{ Mouth part}}_{{{\text{ti}}}} + \, \pi_{{{\text{2i}}}} {\text{ Nose part}}_{{{\text{ti}}}} + \, \pi_{{{\text{3i}}}} {\text{ Eyes part}}_{{{\text{ti}}}} + e_{{{\text{ti}}}} \\ & {\text{Level 2 }}\left( {\text{between persons}} \right){:} \, \pi_{{0{\text{i}}}} = \, \beta_{00} + \, \beta_{{0{1}}} {\text{ Ethnicity Asian}}_{{\text{i}}} + \, \beta_{{0{2}}} {\text{ Ethnicity Latino}}_{{\text{i}}} + \, \beta_{{0{3}}} {\text{ Ethnicity Black}}_{{\text{i}}} + \, \beta_{{0{4}}} {\text{ Ethnicity White}}_{{\text{i}}} + r_{{0{\text{i}}}} \\ & {\text{Level 2 }}\left( {{\text{between persons}},{\text{ interaction}}} \right):\pi_{{{\text{1i}}}} = \beta_{{{1}0}} \\ & \quad + \beta_{{{11}}} {\text{ Ethnicity Asian}}_{{\text{i}}} *{\text{ Mouth part}}_{{\text{i}}} + \, \beta_{{{12}}} {\text{ Ethnicity Latino}}_{{\text{i}}} *{\text{ Mouth part}}_{{\text{i}}} \\ & \quad + \beta_{{{13}}} {\text{ Ethnicity Black}}_{{\text{i}}} *{\text{ Mouth part}}_{{\text{i}}} + \, \beta_{{{14}}} {\text { Ethnicity Mixed}}_{{\text{i}}} *{\text{ Mouth part}}_{{\text{i}}} \\ & \quad + \beta_{{{21}}} {\text{ Ethnicity Asian}}_{{\text{i}}} *{\text{ Nose part}}_{{\text{i}}} + \, \beta_{{{22}}} {\text{ Ethnicity Latino}}_{{\text{i}}} *{\text{ Nose part}}_{{\text{i}}} \\ & \quad + \beta_{{{23}}} {\text{ Ethnicity Black}}_{{\text{i}}} *{\text{ Nose part}}_{{\text{i}}} + \, \beta_{{{24}}} {\text{ Ethnicity Mixed}}_{{\text{i}}} *{\text{ Nose part}}_{{\text{i}}} \\ & \quad + \beta_{{{31}}} {\text{ Ethnicity Asian}}_{{\text{i}}} *{\text{ Eyes part}}_{{\text{i}}} + \, \beta_{{{32}}} {\text{ Ethnicity Latino}}_{{\text{i}}} *{\text{ Eyes part}}_{{\text{i}}} \\ & \quad + \beta_{{{33}}} {\text{ Ethnicity Black}}_{{\text{i}}} *{\text{ Eyes part}}_{{\text{i}}} + \, \beta_{{{34}}} {\text{ Ethnicity Mixed}}_{{\text{i}}} *{\text{ Eyes part}}_{{\text{i}}} + r_{{{\text{1i}}}} + r_{{{\text{2i}}}} + r_{{{\text{3i}}}} \\ \end{aligned}$$

We used *R*^2^_GLMM_^68,69^ as a measure of explained variance, which can be interpreted like the traditional *R*^2^ statistic in regression analyses. *R*^2^_conditional_ represents the proportion of variance explained by both fixed and random factors. It has proven to be a useful and reliable estimate in applied work and simulation studies^70–72^. Furthermore, we report Ω^2***^^73–75^, which is a more conservative but conceptually similar measure of overall explanatory power compared to *R*^2^. Of note, Ω^2^ corrects the overestimation of *R*^2^ for population parameters, often resulting in somewhat smaller, more conservative—and less biased—estimates^76,77^. Additionally, following Nakagawa and Schielzeth^69^, we also included AIC and BIC as information criteria indices. The anonymized data as well as all analysis scripts (R script, SPSS-Syntax) are accessible on the Open Science Framework (https://osf.io/uqhr8/?view_only=66c9db9862f3485eb02e244e54914fd2).

### Ethical approval

The Commission for Scientific Integrity and Ethics of the Karl Landsteiner University of Health Sciences, Austria, approved of the proposed study protocol (EK Nr: 1012/2021). The research complies with all relevant ethical regulations (i.e., national and international guidelines (e.g., Declaration of Helsinki)) and Informed Consent was obtained from all raters prior to the study beginning. Raters received a set remuneration of approximately £2.00 upon completion of the study.

## Results

### Descriptive analyses

Approximately three out of four raters (76.7%) indicated that their self-reported ethnicity matched the dominant ethnicity of their environment (see Table S1 in the Online Supplement for ethnicity-specific rates). When asked to identify targets’ ethnicities, raters correctly identified targets’ ethnicities approximately 80% of the time. In so doing, raters achieved the highest accuracy in correctly identifying the ethnicities of White (91.2%) and Black targets (89.1%), followed by Asian (79.9%) and Latino (59.5%) targets. Consistent with the findings on rating difficulty described below, the rates of correct ethnicity identification were similarly high for full faces (90.2%) and eyes part stimuli (86.0%), while the rates for mouth (76.7%) and mid-face (66.7%) stimuli were lower.

Descriptive analyses showed that on a scale from 1 (*not at all difficult*) to 9 (*very difficult*), raters found it easiest to rate the full faces (*M* = 3.1, *SD* = 2.1). Regarding individual facial parts, raters found it more challenging to rate eyes part stimuli when compared to full faces (*M* = 4.5, *SD* = 2.1; *t* = 27.15, *df* = 6728, *p* < 0.001, Cohen’s *d* = 0.66), whereas raters found it substantially more difficult to infer targets’ trustworthiness from mouth stimuli (*M* = 6.4, *SD* = 2.1; reference full faces*, t* = 65.74, *df* = 6734, *p* < 0.001, Cohen’s *d* = 1.60) and mid-face stimuli (*M* = 7.2, *SD* = 1.9; reference full faces, *t* = 83.01, *df* = 6725, *p* < 0.001, Cohen’s *d* = 2.02).

### Trustworthiness of singular facial parts vs. whole faces (RQ1)

**Figure 3:**
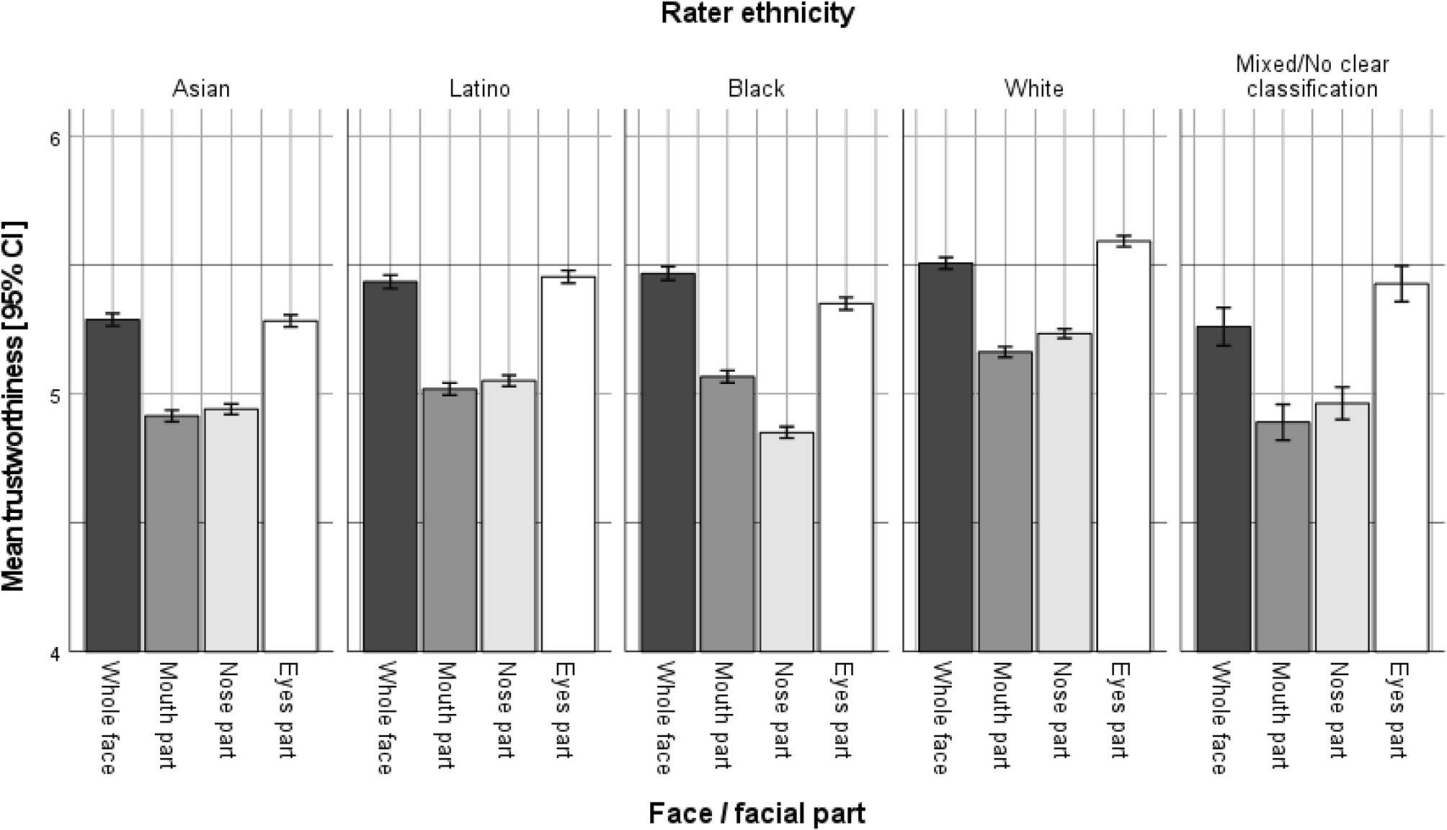
Illustration of RQ1: mean trustworthiness ratings by stimulus type and rater ethnicity.

The overall mean trustworthiness rating for the whole face stimuli (intercept) was moderate, as evidenced by responses being slightly above the mid-point of the scale (5.42 on the 9-point Likert scale). The trustworthiness ratings of eyes-only stimuli did not differ significantly from the full-face ratings (Table 4). When looked at by ethnicity (Fig. 3), trustworthiness ratings for the eyes part still did not significantly differ from the whole face for Asian and Latino raters, and differed only slightly for Black, White, and mixed-ethnicity raters (see Table S5 in the online supplement). The effect sizes had no consistent direction, were rather small, (− 0.03 to + 0.04) compared to the other facial parts (− 0.08 to − 0.14) and thus likely reflect random error.

Overall, trustworthiness judgments formed from the eyes (*M* = 5.42, *SD* = 1.93) did not significantly vary from trustworthiness judgments made from the whole face (*M* = 5.43, *SD* = 2.07), suggesting there was no information loss as a result of occluding the mid-face and mouth (see Table 4). In contrast, mouth (*M* = 5.04, *SD* = 1.87) and mid-face (*M* = 5.02, *SD* = 1.70) stimuli yielded significantly lower mean trustworthiness ratings (for inter-correlations, see Table S4).

### Exploratory analyses (EA)

With respect to EA1, as shown in Table 4 and Figure S2 of the Online Supplement, we found that—on average—male targets received a 0.43 point lower trustworthiness rating on the 9-point Likert-type scale than female targets. Regarding EA2, higher trustworthiness ratings were observed when rater’s and target’s sex were the same (0.09-point increase on 9-point scale, *p* < 0.001) or the eye color matched (0.59-point increase on 9-point scale, *p* < 0.001; see also Figures S3 and S4 in the Online Supplement). Interestingly, matching hair color led to lower trustworthiness ratings (0.09-point decrease on 9-point scale) although the effect was of very modest size. Regarding EA3, we found that the stimulus type-specific difficulty of the ratings did not substantially alter the trustworthiness ratings, and the only significant effect found was very small in size: that is, trustworthiness ratings were 0.04 points higher if mid-face difficulty ratings rose by 1.

More detailed analyses, including additional results and illustration of cross-level interactions between stimulus type (whole face versus parts) and rater ethnicity are provided in Table S5 and Figure S5 in the Online Supplement.

### Trustworthiness of faces/facial parts as a function of rater/target ethnicity (RQ2A) and dominant ambient ethnicity (RQ2B)

**Figure 4:**
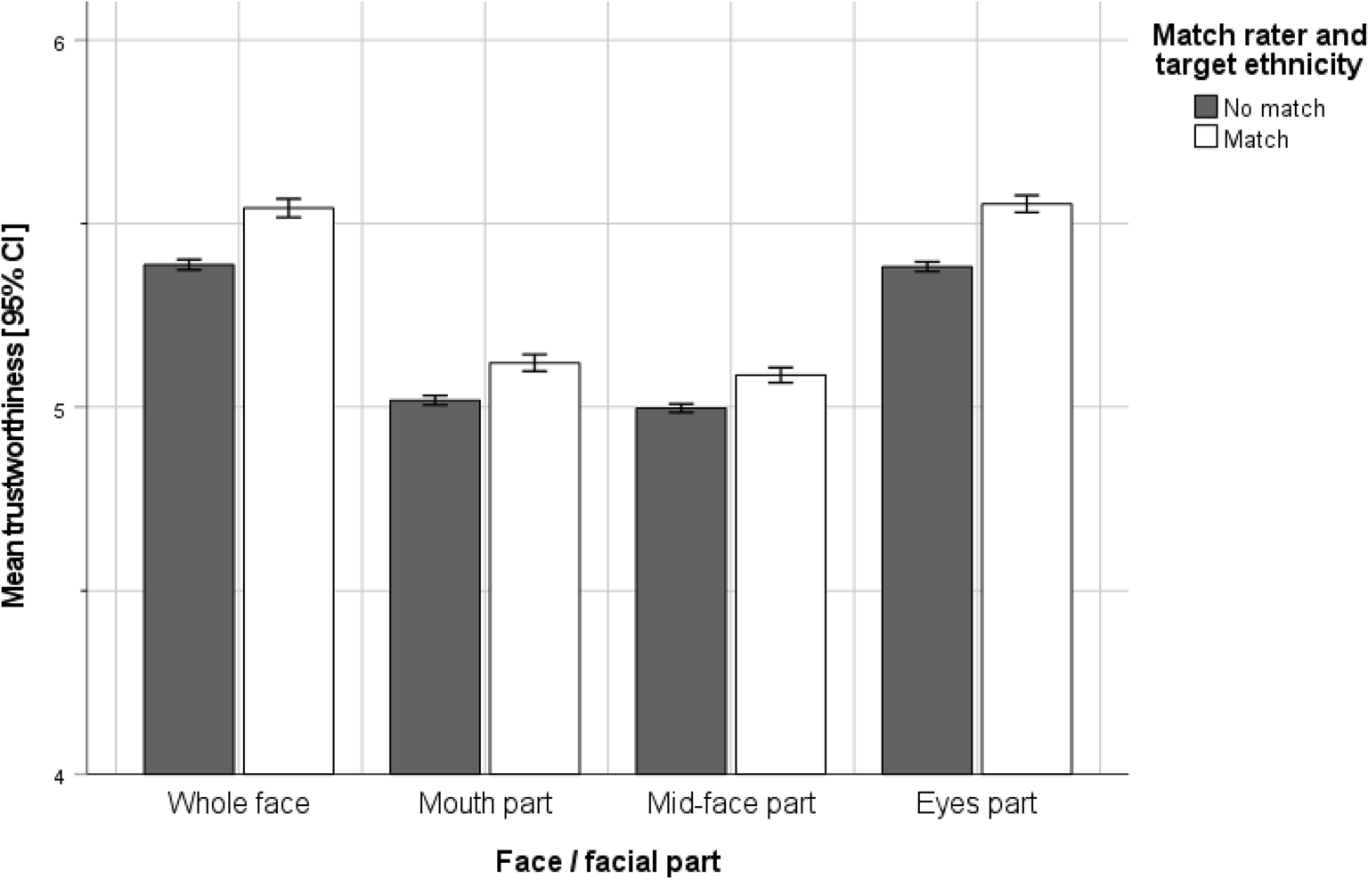
Trustworthiness ratings for targets with rater’s ethnicity (match) vs. targets of different ethnicity (no match).

As can be seen in Table 5 and Fig. 4, independent of whether the whole face was seen or just facial parts, trustworthiness ratings were significantly higher when the raters and target ethnicity did (versus did not) match. Although in general the effects were significant for every stimulus type but with rather low effect size (see Table 5, standardized *B* ranging from 0.03 to 0.05), effects were larger for the whole face (**∆***M* = 0.15**;** *M* = 5.54, *SD* = 2.10 vs. *M* = 5.39, *SD* = 2.06) and the eyes-part (**∆***M* = 0.17**;** *M* = 5.55, *SD* = 1.92 vs. *M* = 5.38, *SD* = 1.93), compared to the mid-face part (**∆***M* = 0.09**;** *M* = 5.09, *SD* = 1.70 vs. *M* = 5.00, *SD* = 1.70) and mouth-part (**∆***M* = 0.10**;** *M* = 5.12, *SD* = 1.89 vs. *M* = 5.02, *SD* = 1.86).

**Figure 5:**
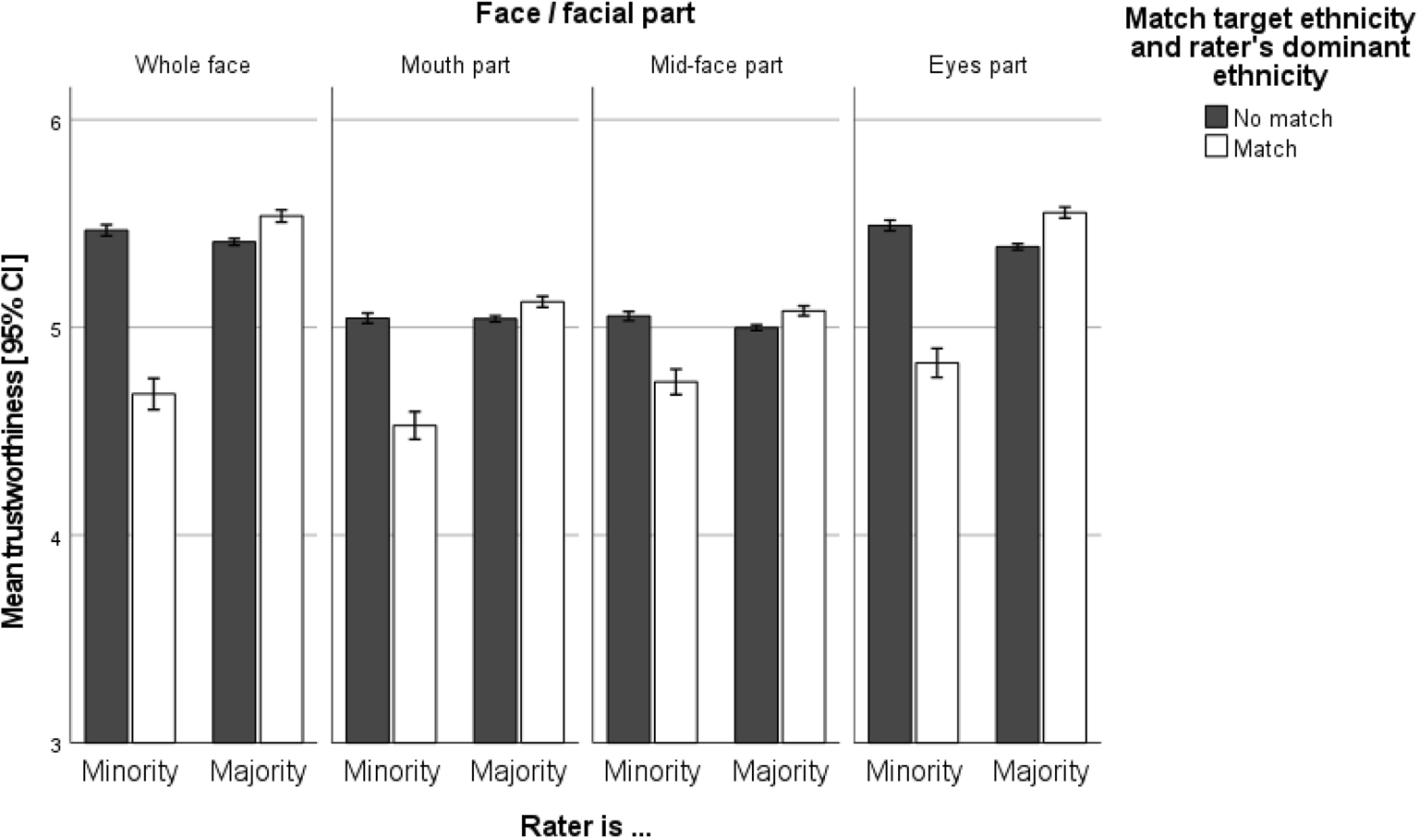
Illustration of RQ2B: mean trustworthiness ratings by stimulus type and rater status (ethnic majority versus ethnic minority).

Furthermore, we analyzed trustworthiness ratings depending on whether the target stimuli matched the dominant ethnicity of the rater (RQ2A) and whether this was influenced by whether raters themselves were from the dominant ethnicity (i.e., majority) or not (i.e., minority, e.g., due to immigration), as described in RQ2B. As can be seen from Table 6 and Fig. 5, again regardless of whether raters judged the whole face or facial parts, the (mis-)match of rater’s dominant ambient ethnicity and target’s ethnicity did not influence the trustworthiness ratings, except for a tiny, albeit significant, effect on the eyes part (standardized *B* = 0.01, *p* < 0.05). Importantly, as a second part of RQ2, we also examined whether the observed effects depended on the match (or lack thereof) between raters’ ethnicity and their dominant ambient ethnicity—in other words whether or not they are part of the ethnic minority in their living environment. Indeed, as can be seen in Fig. 5 (and as is also captured by the significant interaction effects exhibited in Table 6), when raters belonged to an ethnic *majority*, trustworthiness ratings were higher (i.e., for whole face stimuli: standardized *B* = 0.20, *p* < 0.001) for stimuli depicting the same ethnicity (versus stimuli from other ethnicities). In contrast, when raters belonged to an ethnic *minority*, trustworthiness ratings were substantially lower for stimuli depicting members of the ethnic majority (i.e., the dominant ethnicity in the rater’s environment) compared to stimuli depicting members of other ethnicities (including the rater’s ethnicity). The effect size of this interaction effect was—once again—slightly larger for the whole face and eyes region (standardized *B* = 0.20, and 0.21, respectively) than the mouth and mid-face region (0.17 and 0.13, respectively).

## Discussion

The present registered report sheds new light on the psychological mechanisms underlying social face evaluation. Leveraging a global, ethnically diverse, and large-scale sample, we found that the single most informative source of information for humans’ trustworthiness ratings of faces are the eyes and eyebrows (RQ1). This is supported by our empirical finding that, across all raters, trustworthiness ratings based on targets’ eyes and eyebrows did not differ substantially from the full-face ratings (i.e., there was no information gain or loss for raters when looking at the whole face vis-à-vis only the eyes and eyebrows). Consistent with past research on ingroup biases (e.g.,^45,50^), raters of all ethnicities rated faces/facial parts of targets from their own ethnicity as significantly more trustworthy than those of targets from other ethnicities (RQ2A; see Fig. 4). Furthermore, offering additional nuance, our investigation of RQ2 revealed several moderation effects. Specifically, when raters were part of the ethnic majority of their social environment (e.g., natives), targets with matching ethnicity (i.e., also natives) were rated as more trustworthy than targets from other ethnicities (i.e., non-natives). Conversely, when raters were from an ethnic minority (e.g., due to migration), trustworthiness ratings of targets belonging to the ethnic majority (i.e., natives) were substantially lower than trustworthiness ratings of targets belonging to ethnic minorities (i.e., non-native ethnicities, including the rater’s own ethnicity).

It is important to note, that—in absolute terms—most observed effects were small (i.e. RQ2A: β _*range*_ = 0.03–0.05; **∆***M*_*range*_ = 0.09–0.17; RQ2B: all βs ≤ 0.20) to medium in size (i.e., RQ1: **∆***M*_*range*_ = 0.38–0.41). However—consistent with recent theoretical and empirical pushes within the psychological sciences calling to focus evaluations on effect sizes (versus statistical significance) and consider them in context^78–80^—we would like to argue that these effects may nevertheless matter in everyday life. For example, considering that raters judged one and the same target in all stimuli type conditions, even the relatively small differences in mean trustworthiness between full-face and eyes condition versus mid-face and mouth condition (up to 0.41 points on a 9-point scale) may take on direct relevance in everyday social situations when certain facial parts are covered, for instance due to religious beliefs or hygienic reasons—a common global occurrence since the outbreak of the Covid-19 pandemic. Likewise, the similarly-sized gender differences in perceived trustworthiness observed in EA1 (which may be mechanistically explained by humans’ tendency to search for sexually dysmorphic features in human faces^35–37^), may—when considered across hundreds of thousands of individuals—actively contribute to harmful societal phenomena such as the formation of gender stereotypes, prejudices or gender discrimination.

### Eyes as the most important trustworthiness indicator

While the current results do not challenge the extant empirical evidence in support of holistic face perception, they underpin the importance of the eyes-region in social face evaluation, in line with prior research^28–30^. Aligning with previous work demonstrating that humans particularly rely on information from the eyes-region for emotion perception and empathizing with others (e.g.,^27–30^), the current work highlights that social impressions of trustworthiness are also primarily inferred from and informed by inspection of targets’ eyes. Similarly, another important result of the current study is that the relative dominance of the eyes-region as a central source of trustworthiness assessments was found across all rater ethnicities (except for a minor deviation, i.e. trustworthiness ratings from Black raters differed significantly between full face stimuli and eyes stimuli, but with the eyes still being the best indicator), providing additional evidence for the cross-cultural generalizability of trustworthiness evaluation processes. Consistent with the centrality of the eyes-region in forming trustworthiness judgments from faces across ethnicities, raters found it similarly easy to (1) correctly identify targets’ ethnicity and (2) rate the eyes part for trustworthiness compared to full-face stimuli, and this effect was robust across all rater ethnicities. In contrast, the mid-face and mouth stimuli exhibited substantially lower correct ethnicity identification rates and were perceived as more difficult to judge, indicating that the eyes region might be a better cue for ethnicity identification—further cementing their social relevance.

With this in mind, considering that trustworthiness features enable us to make inferences about the harmfulness of other people’s intentions—and can be affected by the appearance of the eyebrows and eye size^14^, future research should test if the size (versus other attributes) of targets’ eyes primarily guides trustworthiness judgments. Specifically, large eyes are perceived as more youthful^14^, and given that children are usually considered harmless in the context of potential physical danger towards one’s own person, perceptions of youthfulness as a result of eye size may contribute to trustworthiness judgments. Alternatively future research should test whether the angle and height of targets’ eyebrows play an especially prominent role in guiding trustworthiness judgments, given that targets with V-shaped eyebrows are perceived as threatening (e.g.,^13,26,81^). For example, trustworthiness judgments of faces are driven by the extent to which a face resembles emotion expressions^15^, and the eyebrows are the most crucial features in communicating emotion^28^. Therefore, although the current work identifies the eyes part as a key facial region guiding trustworthiness judgments, future research is needed to isolate *why* this specific region is so critical.

### Intergroup relations in trustworthiness evaluations

One characteristic of faces and facial parts that this research focused on was the ethnicity of raters and targets, as well as the dominant ethnicity of raters’ social environments. Examining RQ2, we found that the ethnic ingroup-outgroup effect,—that is, preferential treatment of ingroup members—was detected across all stimulus types, and ethnicities, evidenced by raters judging ingroup members as more trustworthy than outgroup members. Taken together, we interpret this to support the position (e.g.,^40,44,50^) that ethnicity-based ingroup preference on the basis of trustworthiness judgments is a widespread social phenomenon, evidenced across cultures, and relevant to face perception.

Relatedly, the findings of our exploratory analyses suggest ingroup-outgroup effects beyond ethnicity. Specifically, we observed that targets who matched raters’ eye color or sex received more favorable trustworthiness ratings, however, this was not the case for natural hair color, where a significant *negative* effect was found although of tiny effect size (standardized *B* = − 0.03; *p* < 0.001). Although sex represents a central social identity category, eye color is also a grouping feature that is related to membership in distinct social group, including race. Therefore, an alternative explanation for the effect of matching eye color on perceived trustworthiness might be the linkage with attractiveness, mating and reproduction, or hedonic fluency as a result of repeated exposure to a particular eye/hair color when it matches the rater’s own eye/hair color^82–84^.

Examining RQ2A and RQ2B revealed that when rater and target ethnicity matched, the dominant ethnicity of rater’s social environment did not significantly influence trustworthiness ratings. However, when target and rater ethnicity did not match, two different scenarios were identified: (A) If, additionally, raters’ own ethnicity and the dominant ethnicity of their social environment matched, trustworthiness ratings for targets decreased. In contrast, (B) when raters’ own ethnicity and the dominant ethnicity of their social environment did not match, targets were rated almost equally as trustworthy as if the target was of the rater’s own ethnicity (i.e., ethnic ingroup). Therefore, the ingroup/outgroup classification of targets seems to be based on the rater’s own ethnicity, rather than the ethnicity of their social environment. As one’s own ethnicity represents the point of reference, it appears plausible that the shared feature of raters considering themselves as an outgroup member in their environment (no match with social environment) and targets being considered as an outgroup (no match with raters’ ethnicity), may in fact induce a perception of community and camaraderie (both outgroup members). Stated differently, individuals may perceive outgroup members as ingroup members joined together by similarly being outgroup members in a separate domain. This, in turn, makes the target, which is an ethnic outgroup member (reference: participant ethnicity), an ingroup member due to shared outgroup experiences. Interestingly, this social psychological pattern is consistent across all stimulus types—at least in part thanks to the fact that raters are able to correctly identify targets’ ethnicity based on any facial part, even though accuracy is highest for eyes and full-face stimuli (see^85^ for an elaborate discussion of ingroup biases and self-categorization).

Turning to differences in perceived trustworthiness based on demographic characteristics beyond ethnicity, our results demonstrated that male targets were rated significantly less trustworthy than females. This effect (*B* = − 0.43; standardized *B* = − 0.13) should be put in the context of evolutionarily essential information processing, as *trustworthiness* is associated with the perception of whether someone’s intentions might be dangerous (and should therefore be avoided) or if the person can be approached safely. Additionally, humans evaluate the physical ability and strength to realize potentially harmful actions^4^. Considering that human’s stereotypical mental representation of males consists of strong, muscular, or athletic men, the reduction in trustworthiness ratings for male targets might be due to implicit perceptions of physical strength—that is, the increased potential to realize harmful intentions. Moreover, similar to a halo-effect, raters’ potential ability to draw inferences about target’s sex without seeing the full faces could contribute to this effect. This could be explained with mental representations of sex-specific face morphology (e.g.,^86,87^), which are critically shaped by information based on the mouth (e.g., lips, chin, jaw) and eyes part (e.g., eyebrows).

### Limitations and future directions

The results of the present study should be interpreted within the broader context of transnational, cross-cultural, multi-ethnic studies with all of the advantages and challenges that come with such endeavors. As such, the big benefit of worldwide participant databases, like *Prolific*, is, that we can reach an ethnically-diverse globe-spanning sample (for a detailed geographic breakdown see Figure S1 in the Online Supplement). However, we note that some regions (e.g., Asia, Africa) are still underrepresented—which is also seen in the current study, where despite the availability of Mandarin and Japanese-language versions, only comparatively small numbers of Asian raters living in Asia could be recruited. We also acknowledge that even though we successfully recruited the required participant number for each subsample, the number of eligible Asian raters actually living in Asia was small, thus limiting the generalizability of our findings. This shortcoming could be addressed by combining different participant databases (focusing on different world regions, e.g., Asia, Europe etc.) or through a collaborative multi-site multi-lab effort^88,89^.

Another limitation lies in the spontaneity of the trustworthiness ratings. In the current study, raters were instructed to gauge target’s trustworthiness as spontaneously as possible and an inspection of item-specific response times across the whole sample, suggests that this is indeed what raters did. Nevertheless, it would be interesting for future studies to implement limited exposure and response times (i.e., response-window design) as well as experimental settings (e.g., face-to-face). With respect to the current study’s design, it should be highlighted that the targets used only covered a narrow age range spanning from 25 to 35 years, which should be broadened in future research. Moreover, we would like to note that the selected stimuli sets used in this study were exclusively composed of targets that were rated as moderately trustworthy. To consolidate and extend our current work, future research may thus seek to investigate stimuli sets with normally distributed trustworthiness ratings (assuming trustworthiness ratings in the population are normally distributed), which would further allow targeted examinations of stimuli with extreme trustworthiness ratings.

Analogous to targets’ age, a follow-up study may also expand the age range of raters by deliberately recruiting younger raters, especially infants. That way, one could examine whether the social face evaluation mechanisms and processes observed here are likely to be innate or likely to emerge at certain developmental stages. Taken together, while the current research made special efforts to ascertain cross-cultural generalizability, future work is needed to assess the generalizability of face perception across age. Neuropsychologically speaking, follow-up research should investigate the precise cerebral processes and linkages determining the evaluation of social information (e.g., ingroup vs. outgroup) as well as evolutionarily relevant information (e.g., trustworthy vs. untrustworthy). For this purpose, it might be beneficial to consider closer examination and contrasting of patients with impairments regarding face evaluation/recognition (e.g., prosopagnosia) or patients with mental illnesses/psychiatric disorders (e.g., schizophrenia, avoidant personality disorder, paranoid personality disorder, anxiety disorder).

As the findings of the current study provide empirical evidence that the eyes are used as the most important facial indicator for trustworthiness ratings, future research should be devoted to the question of which anatomic features, muscles, and proportions, in general and particularly in terms of the eyes region, are perceived as more (or less) trustworthy. Faces are often observed in tandem with the body. Yet, much less research addresses how body shapes, sizes, and features, guide and contribute to person perception. Future research is needed to tackle how and why bodies contribute to—or interfere with—trustworthiness judgments.

At an even more basic level, future research might analyze whether the lack of information when seeing only parts of the face might be partly responsible for the patterns observed in the present study. Specifically, it might be that if raters have little information to judge facial parts, their trustworthy ratings are low. That is, the ratings might be a function of information richness, rather than the focus within the face (mouth, mid-face, eyes). Fortunately, we asked participants about how easy it was to judge each facial part (assuming that less information results in higher burden to find valid judgements). In the current sample, all such correlations between judgement difficulty and trustworthiness ratings were of tiny effect sizes (*r*_mouth_ = 0.006, *r*_nose_ = 0.011, *r*_eyes_ = − 0.028), thus suggesting that trustworthiness ratings are in fact not substantially influenced by the amount of information available from the presented face / facial part stimulus.

## Conclusion

The present research makes an important contribution to the scientific literature and our understanding of social perception. Harnessing an ethnically-diverse, global (i.e., 32 countries), and large sample, the current registered report provides empirical evidence that trustworthiness lies not only in the eye of the beholder, but just as much in the eye of the beholden^57^.

## Supplementary Information

Supplementary Information.

## Data Availability

The raw data and materials can be accessed via https://osf.io/uqhr8/?view_only=66c9db9862f3485eb02e244e54914fd2.

## Code availability

The materials can be accessed via https://osf.io/uqhr8/?view_only=66c9db9862f3485eb02e244e54914fd2.
