# Supplementary material for: Registered report: Social face evaluation: ethnicity-specific differences in the judgement of trustworthiness of faces and facial parts
Source: Sci Rep. 2022 Oct 31;12:18311. doi: 10.1038/s41598-022-22709-9 (PMC9622746; doi:10.1038/s41598-022-22709-9)

Registered Report: Social face evaluation: Ethnicity-specific differences in the judgement of trustworthiness of faces and facial parts

Online Supplement

Sample characteristics

Table S1. Rater characteristics.

Table S2. Difficulty ratings by stimulus type and ethnicity.

Table S3. Ethnicity check question: Correct identification rates.

Table S4. Pearson correlations of trustworthiness ratings between full face and facial parts stimuli.

Figure S1. Geographic distribution of study sample.

Main Analyses

Figure S2. Mean trustworthiness by stimulus type and target sex.

Figure S3. Illustration of RQ1 + EA2 (matching sex).

Figure S4. Illustration of RQ1 + EA2 (matching eye color).

Cross-level interactions

Table S5. Trustworthiness assessments of whole face (reference category) and different facial parts by rater ethnicity (RQ1 – cross-level interaction).

Figure S5. Illustration of RQ1 + effects by rater ethnicity.

Table S1. Rater characteristics.

		Final sample<br>*n* = 3,371	Asian<br>*n* = 743	Black<br>*n* = 908	Latino<br>*n* = 731	White<br>*n* = 891	Mixed<br>*n* = 93
			Categorical variables [%]				
Sex	Female	63.1	61.9	72.5	58.0	58.7	62.4
	Male	36.0	37.3	27.1	40.6	40.4	35.5
	Other	0.7	0.7	0.2	1.1	0.7	2.2
Eye color	Blue	9.9	0.1	0.6	1.8	35.5	0.0
	Green	7.8	0.7	0.2	6.6	22.1	11.8
	Brown	80.7	97.3	98.3	91.5	39.4	88.2
	Grey	1.4	1.6	0.7	0.1	3.0	0.0
Hair color	Blonde	4.7	0.0	0.2	1.2	16.5	1.1
	Brown	41.5	15.5	19.1	59.5	69.0	62.4
	Black	52.2	84.1	80.4	38.6	9.5	35.5
	Red	1.1	0.0	0.2	0.1	3.7	0.0
	Other	0.6	0.4	0.1	0.5	1.2	1.1
Dominant ambient ethnicity	Asian	11.9	52.9	0.2	0.1	0.2	3.2
	Black	24.7	1.6	87.7	0.3	0.8	15.1
	Latino	17.4	0.4	0.1	75.8	2.0	10.8
	White	33.3	24.2	2.5	12.6	90.0	25.8
	Mixed	12.6	20.9	9.4	10.9	7.0	45.2
Language version	English	86.7	99.3	100.0	59.0	85.0	90.3
	German	0.8	0.0	0.0	0.0	2.9	0.0
	Spanish	12.4	0.0	0.0	41.0	12.1	9.7
	Japanese	0.1	0.0	0.0	0.0	0.0	0.0
	Mandarin	0.0	0.7	0.0	0.0	0.0	0.0
			Metric variables [years]				
Age	Mean (SD)	30.5 (11.1)	29.6 (9.8)	27.1 (5.5)	26.4 (8.0)	38.4 (14.1)	28.1 (10.3)

Education	Mean (SD)	14.61 (4.7)	15.7 (4.0)	11.9 (5.7)	15.5 (3.6)	15. (3.7)	13.9 (4.8)
Matching ethnicity of participant and participants' social environment [%]							
Ident Ethnicity rate		76.7	52.9	87.7	75.8	90.0	45.2

Table S2. Difficulty ratings by stimulus type and ethnicity.

	Mean Difficulty Full faces (SD)	Mean Difficulty Eyes (SD)	Mean Difficulty Mid-face (SD)	Mean Difficulty Mouth (SD)
Overall sample	3.1 (2.1)	4.5 (2.1)	7.2 (1.9)	6.4 (2.1)
Asian	3.2 (2.1)	4.5 (2.1)	7.0 (2.0)	6.4 (2.1)
Black	2.9 (2.1)	5.1 (2.2)	7.2 (2.0)	6.7 (2.2)
Latino	2.9 (2.1)	3.7 (1.9)	7.0 (2.0)	6.1 (2.1)
White	3.3 (2.0)	4.5 (1.9)	7.3 (1.8)	6.4 (1.8)
Mixed	3.0 (2.0)	4.2 (2.1)	7.0 (2.1)	6.1 (2.0)

Table S3. Ethnicity check question: Correct identification rates in %.

	Mean Overall ethnicity identification rate (SD)	Mean Full faces ethnicity identification rate (SD)	Mean Eyes ethnicity identification rate (SD)	Mean Mid-face ethnicity identification rate (SD)	Mean Mouth ethnicity identification rate (SD)
All stimuli	79.9 (21.6)	90.2 (12.2)	86.0 (16.5)	66.7 (26.1)	76.7 (21.6)
Asian targets	79.7 (20.6)	95.5 (3.7)	97.7 (0.7)	63.0 (11.7)	62.7 (20.6)
Black targets	89.1 (19.5)	96.7 (6.5)	90.0 (13.6)	76.8 (31.6)	93.1 (14.2)
Latino targets	59.5 (17.4)	74.8 (9.0)	63.6 (7.1)	39.9 (16.6)	59.8 (14.2)
White targets	91.2 (12.5)	94.0 (12.8)	92.6 (13.3)	87.0 (13.2)	91.2 (12.0)

Table S4. Pearson correlations of trustworthiness ratings between full face and facial parts stimuli.

	1.	2.	3.
1. Full face			
2. Eyes part	.69		
3. Nose part	.56	.66	
4. Mouth part	.66	.68	.73

Note. All correlations *p* <.001.

Figure S1. Geographic distribution of study sample.

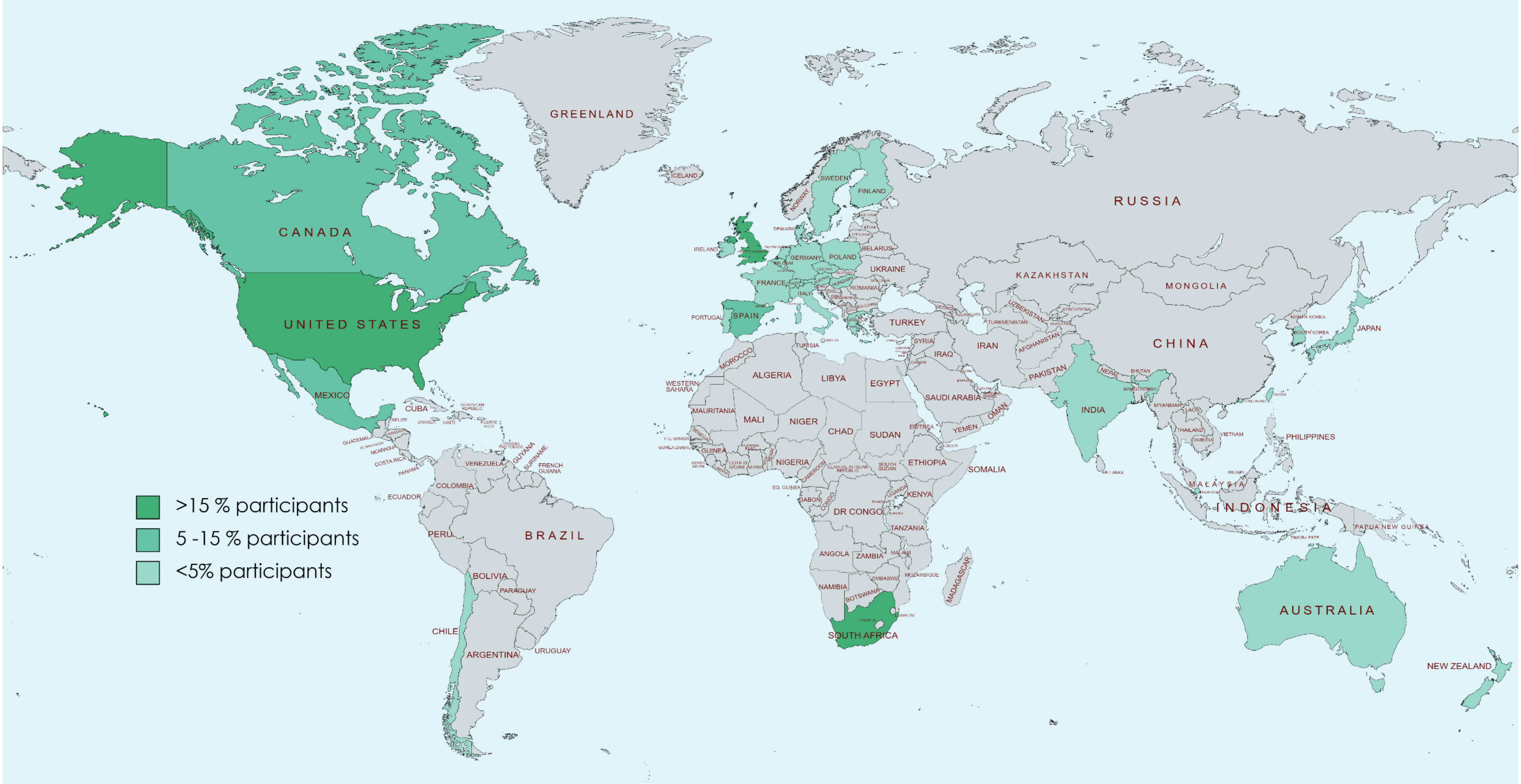

Note. Figure S1 was created by the authors with the open-source software mapchart.net (https://www.mapchart.net/world.html; created on February, 17th, 2022)

Figure S2. Mean trustworthiness by stimulus type and target sex.

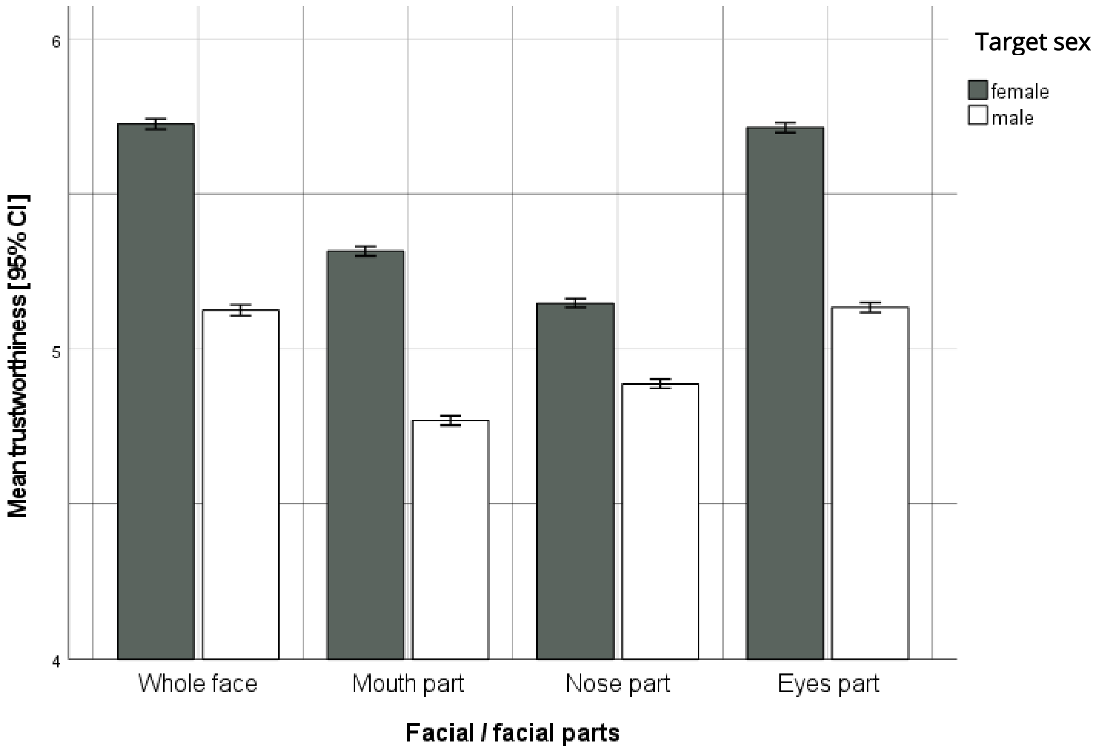

Figure S3. Mean trustworthiness by stimulus type and rater-target sex match.

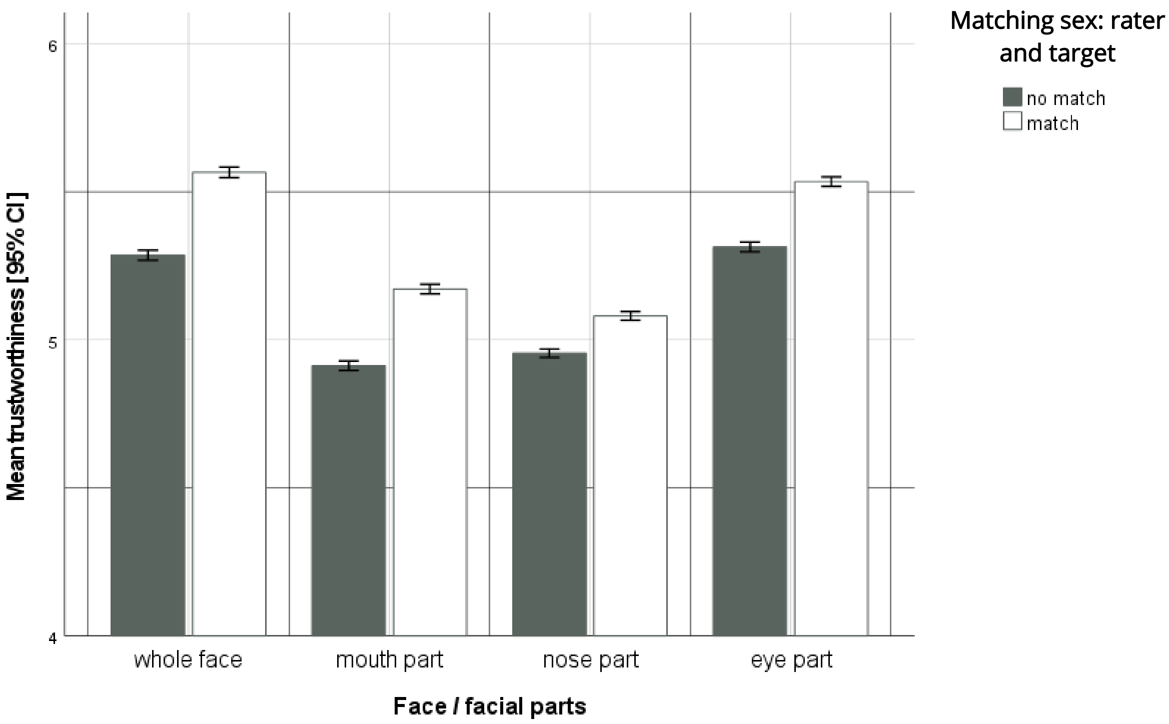

Figure S4. Mean trustworthiness by stimulus type and rater-target eye color match.

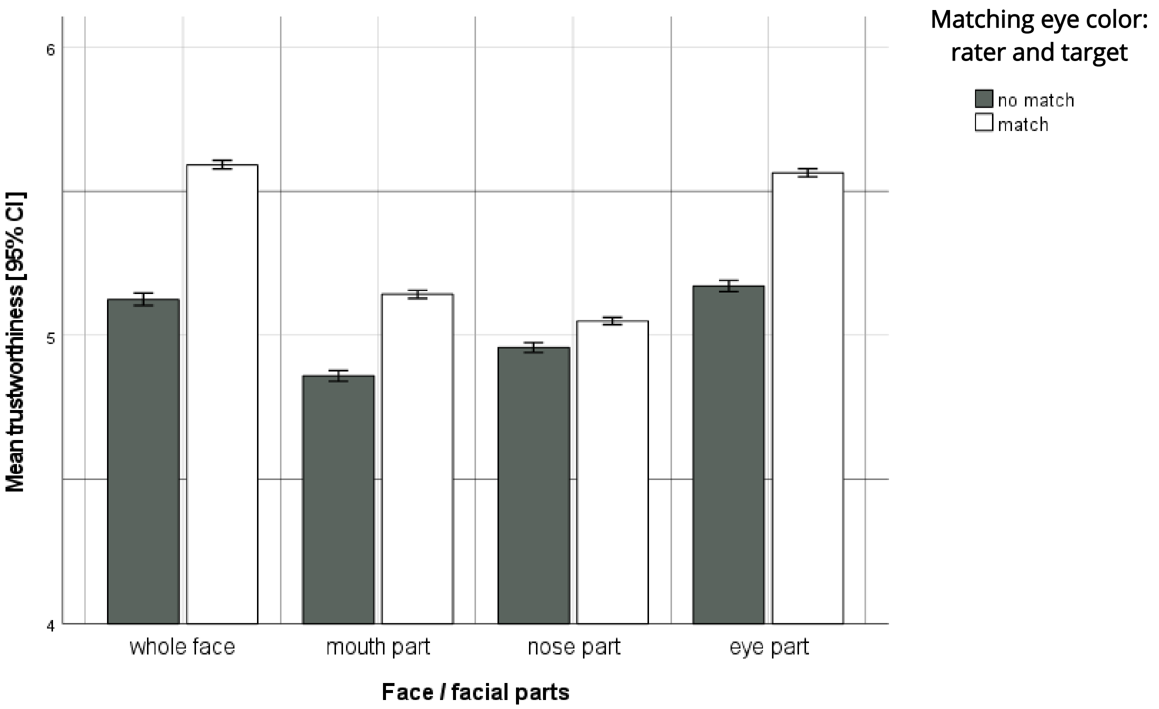

Cross-level interactions

The results of the cross-level interactions are shown in Table S4. In general, even though the cross-level interaction model included more predictors than the model fitted to test RQ1, the amount of explained variance did not change substantially ($R^2_{conditional}$ 33% for both models, $R^2_{marginal}$ was 0.4% higher for cross-level interaction model), as Level 2 variables had low impact with small effect sizes.

The eyes part differed lightly from the full-face ratings, but this effect can be considered extremely small (B = 0.09, p < .001). To this effect may have contributed that especially White participants judged the eyes part as even more trustworthy than the full-face stimuli (see Figure S3 above). Moreover, Asians and participants of mixed ethnicity perceived the targets as significantly less trustworthy compared to the White subsample. However, caution is warranted when drawing such inferences due to the small size of the mixed ethnicity subsample (2.8% of all participants; n = 93) which resulted in large confidence intervals.

Regarding the interactions of participant ethnicity and stimulus type, analyses revealed that interactions were non-significant or of very small effect size except for Black participants rating the mid-face and eyes stimuli (see Figure S3 above). More specifically, if a Black participant rated mid-face (B = -0.34, p < .001) or eyes stimuli (B = -0.20, p < .001), the effect of rater ethnicity on trustworthiness ratings got stronger (i.e., in this case more negative), indicating that the trustworthiness ratings of these stimulus types deviate more strongly from the full-face ratings.

Table S4. Trustworthiness assessments of whole face (reference) and different facial parts depending on participants' ethnicity (RQ1 – cross-level interaction).

	Fixed					Random	
	Coeff.	*B*	*CI*	*SE*	*t*	Coeff.	*SD*
Intercept (Reference)	β_{00}	5.51	5.44 – 5.58	0.04	153.0***	r_{0i}	1.04
Within-person (reference whole face)							
Mouth part	β_{10}	-0.34	-0.39 – -0.30	0.02	-14.3***	r_{1i}	0.60
Nose part	β_{20}	-0.27	-0.33 – -0.22	0.03	-10.2***	r_{2i}	0.70
Eyes part	β_{30}	0.09	0.05 – 0.12	0.02	4.3***	r_{3i}	0.45
Between-person (reference White)							
Ethnicity Asian	β_{01}	-0.22	-0.32 – -0.11	0.05	-4.1***		
Ethnicity Latino	β_{02}	-0.07	-0.18 – 0.03	0.05	-1.4		
Ethnicity Black	β_{03}	-0.04	-0.14 – 0.06	0.05	-0.8		
Ethnicity Mixed	β_{04}	-0.25	-0.48 – -0.02	0.12	-2.1*		
Interaction							
Mouth:Asian	β_{11}	-0.03	-0.10 – 0.04	0.04	-0.82		
Mouth:Latino	β_{12}	0.04	-0.14 – -0.00	0.04	-1.99*		
Mouth:Black	β_{13}	0.03	-0.12 – 0.01	0.03	-1.65		
Mouth:Mixed	β_{14}	0.08	-0.18 – 0.13	0.08	-0.33		
Nose:Asian	β_{21}	-0.07	-0.15 – 0.00	0.04	-1.87		
Nose:Latino	β_{22}	-0.11	-0.19 – -0.03	0.04	-2.78**		
Nose:Black	β_{23}	-0.34	-0.42 – -0.27	0.04	-9.10***		
Nose:Mixed	β_{24}	-0.02	-0.20 – 0.15	0.09	-0.28		
Eyes:Asian	β_{31}	-0.09	-0.15 – -0.03	0.03	-3.03**		
Eyes:Latino	β_{32}	-0.07	-0.12 – -0.01	0.03	-2.21*		
Eyes:Black	β_{33}	-0.20	-0.26 – -0.15	0.03	-7.17***		
Eyes:Mixed	β_{34}	0.08	-0.05 – 0.21	0.07	1.25		
$R^2_{conditional}$ = 33%, $R^2_{marginal}$ = 1.4%, AIC = 1631865, BIC = 1632205, Ω^2 = 34%							

Note. Reference category was the trust ratings of the whole face. *p < .05, **p < .01, ***p < .001. Ω^2 is another effect size measure that can be interpreted as the share variance accounted for by the overall model.

Figure S5. Illustration of RQ1 + effects by rater ethnicity.

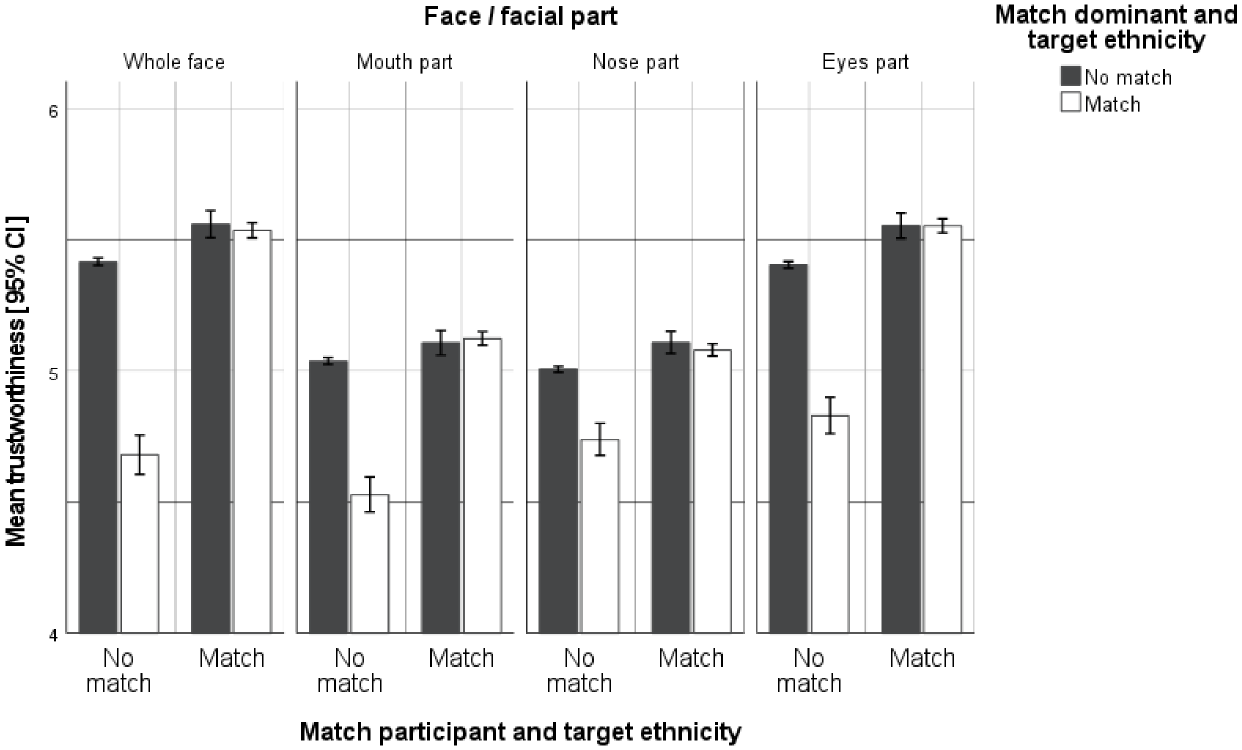

Supplement: Supplementary file 1 — Supplementary Information. [file 41598_2022_22709_MOESM1_ESM.pdf]
